# Regulation of Sexually Dimorphic Expression of Major Urinary Proteins

**DOI:** 10.3389/fphys.2022.822073

**Published:** 2022-03-31

**Authors:** Dustin J. Penn, Sarah M. Zala, Kenneth C. Luzynski

**Affiliations:** Department of Interdisciplinary Life Sciences, Konrad Lorenz Institute of Ethology, University of Veterinary Medicine Vienna, Vienna, Austria

**Keywords:** major urinary proteins, MUP, house mice, *Mus musculus*, pheromones, sexual selection, differential sex gene expression, sexual dimorphism

## Abstract

Male house mice excrete large amounts of protein in their urinary scent marks, mainly composed of Major Urinary Proteins (MUPs), and these lipocalins function as pheromones and pheromone carriers. Here, we review studies on sexually dimorphic MUP expression in house mice, including the proximate mechanisms controlling MUP gene expression and their adaptive functions. Males excrete 2 to 8 times more urinary protein than females, though there is enormous variation in gene expression across loci in both sexes. MUP expression is dynamically regulated depending upon a variety of factors. Males regulate MUP expression according to social status, whereas females do not, and males regulate expression depending upon health and condition. Male-biased MUP expression is regulated by pituitary secretion of growth hormone (GH), which binds receptors in the liver, activating the JAK2-STAT5 signaling pathway, chromatin accessibility, and MUP gene transcription. Pulsatile male GH secretion is feminized by several factors, including caloric restriction, microbiota depletion, and aging, which helps explain condition-dependent MUP expression. If MUP production has sex-specific fitness optima, then this should generate sexual antagonism over allelic expression (intra-locus sexual conflict) selectively favoring sexually dimorphic expression. MUPs influence the sexual attractiveness of male urinary odor and increased urinary protein excretion is correlated with the reproductive success of males but not females. This finding could explain the selective maintenance of sexually dimorphic MUP expression. Producing MUPs entails energetic costs, but increased excretion may reduce the net energetic costs and predation risks from male scent marking as well as prolong the release of chemical signals. MUPs may also provide physiological benefits, including regulating metabolic rate and toxin removal, which may have sex-specific effects on survival. A phylogenetic analysis on the origins of male-biased MUP gene expression in *Mus musculus* suggests that this sexual dimorphism evolved by increasing male MUP expression rather than reducing female expression.

## Introduction

“Sexual dimorphism is common throughout the animal kingdom. However, a molecular understanding of how sex-specific traits develop and evolve has been elusive.” Williams and Carroll, 2009, p. 797.

Sexually dimorphic traits are common and expected to evolve when they confer sex-specific effects on survival or reproductive success (Darwinian fitness). Such sex-specific fitness optima are expected to generate intra-locus sexual conflict, a form of sexual antagonism over allelic expression (“conflict over shared genes”; Pennell and Morrow, 2013). Because males and females share most of their genomes, such intra-locus sexual conflict can only be resolved by the evolution of sex-limited or sex-specific gene expression, that is, the repression or gain in gene expression in one sex. Until sexual conflict is completely resolved, sexual dimorphic traits will remain suboptimal for either sex. Investigating hypotheses about sexual dimorphisms at both proximate and evolutionary levels of analysis is challenging. Although the genes and physiological mechanisms controlling the expression of sexual dimorphic traits have been determined in a few model organisms, their adaptive functions and evolutionary origins are still unknown. And although the adaptive functions and evolutionary origins of sexually dimorphic traits have been studied in many non-model species, the molecular mechanisms controlling their expression are rarely known. The simplest route to addressing this challenge is to determine the adaptive functions and evolutionary origins of sexually dimorphic traits in model organisms—and their wild counterparts—rather than trying to identify the genes and proximate mechanisms controlling sexually dimorphic traits in non-model organisms (Badyaev, 2002; Williams and Carroll, 2009).

**Figure 1:**
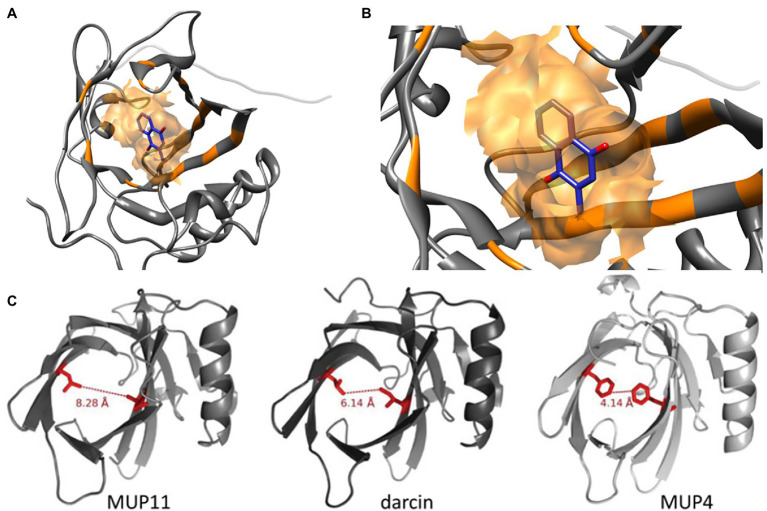
Molecular Structures of MUP Proteins and Pheromone Ligands. MUPs are low molecular weight (18–40 kDa) proteins (162 amino acids) in the structural family of lipocalins, which have a barrel-shaped tertiary structure used for binding and transporting small molecules (Diez-Hermano et al., 2021). **(A)** The models shown here illustrate how each MUP contains a cavity that binds small hydrophobic ligands **(B)**, for example, menadione from Ricatti et al. (2019). These small molecules include male pheromones, such as 2-s-butyl-4,5-dihydrothiazole, 3,4-dehydro-exobrevicomin, and 6-hydroxy-6-methyl-3-heptanone (Böcskei et al., 1992; Žídek et al., 1999; Timm et al., 2001; Mucignat-Caretta and Caretta, 2014). **(C)** The binding cavities and interatomic distances between two similar residues are shown in red for a central MUP proteoform (MUP11), and the peripheral proteoforms of darcin and MUP4. MUP20 shows binding specificity for the pheromone 2-s-butyl-4,5-dihydrothiazole (SBT). Figure used with permission from Phelan et al. (2014).

Here, we provide an integrative review of studies on sexually dimorphic expression of Major Urinary Proteins (MUPs) in house mice (*Mus musculus*; Figure 1). MUP genes are mainly expressed in the liver, they are the most highly expressed genes in the liver, and from the serum, MUPs are excreted in urine. MUP expression is a sexually dimorphic trait, and male mice excrete 2–8 times more protein in their urine than females. The molecular mechanisms controlling this sexual dimorphism are complex and provide a fascinating example of how the brain uses endocrine signals secreted by the pituitary gland to control the expression of genes in the liver and other target organs (Holloway et al., 2008). MUPs are also expressed in several secretory tissues, however, aside from lachrymal glands and nasal secretions, their expression is not sexually dimorphic (Shaw et al., 1983) and their functions are still unclear (Stopková et al., 2021). In contrast, the chemical signaling functions of urinary MUPs have been studied for many years. In males, MUPs bind and transport volatile pheromones, and they stabilize their evaporation from urinary scent marks (Robertson et al., 1993; Hurst et al., 1998; Cavaggioni et al., 2006, 2008). Through this time-release mechanism, MUPs are expected to prolong the influence of volatile male pheromones on conspecifics. Some MUPs also act as pheromones themselves, activating sensory neurons in the vomeronasal organ (VNO) and eliciting aggressive behavior from males (Mucignat-Caretta et al., 2004; Chamero et al., 2007, 2011; Kaur et al., 2014) and maternal aggression (Martín-Sánchez et al., 2015). For example, MUP20 (“darcin”) increases female attraction to male versus female urinary scent (Roberts et al., 2010, 2012). Females are attracted to male urine spiked with MUPs during estrus when MUP-detecting sensory neurons are expressed (Dey et al., 2015). MUP20 in male urine also influences female behavior by inducing spatial learning (Roberts et al., 2010, 2012) and stimulating neural growth in their brain (Hoffman et al., 2015; Demir et al., 2020). Male scent marks and their male chemical components thus provide an interesting example of a sexually dimorphic extended phenotype and a male chemical signal that influences the physiology, brain, and behavior of females.

In addition to reviewing sexually dimorphic MUP expression in house mice, we also examine questions regarding (1) the proximate mechanisms that control hepatic MUP gene expression (physiology), (2) the development of these mechanisms (ontogeny), (3) their selective maintenance (adaptive functions), and (4) evolutionary origins (phylogeny; “Tinbergen’s Four Questions”). Addressing these questions requires considering processes that operate over vastly different time scales and levels of biological organization (molecules cells, individuals, populations, and species). Before examining sex differences in MUP expression, we first provide more background on house mice and their MUPs.

## Background: On Mice and Their MUPs

### *Mus musculus* Versus *Mus laboratorius*

Studies on MUPs and other chemical signals have mainly been conducted with domesticated, laboratory mouse strains, but these results do not always generalize to wild mice (*nota bene:* “wild type” laboratory mice, outbred strains, and wild-derived strains are not wild mice). Laboratory mice evolved under artificial selection in captivity, they are highly inbred, and they carry a variety of deleterious genes that cause neural, visual, auditory, and epithelial defects (Wahlsten, 1982; Sibilia and Wagner, 1995; Chang et al., 2013). Their behavior, sensory systems, physiology, immune system, and many other traits dramatically differ from their wild counterparts (Smith et al., 1994; Abolins et al., 2017). Laboratory mice are genomic mixtures of three *M. musculus* subspecies, derived mainly from *M. musculus domesticus*, though there is still some debate over their relative contributions. For these reasons, some propose that laboratory mice should be classified as a different species (e.g., *M. laboratorius* or *M. gemisch*; Didion and de Villena, 2013). Regardless, making conclusions about the MUPs or other traits of wild *Mus musculus*, their proximate mechanisms, adaptive functions, and evolutionary origins, require studies on *M. musculus*, and preferably in natural or seminatural conditions.

Wild male house mice are highly territorial and dominant males mark their territories with urinary scent marks. Males produce more scent marks than females and dominant males mark more than subordinates (Desjardins et al., 1973). Males increase scent marking in response to encountering females or female scent (Zala et al., 2004; Lehmann et al., 2013), and scent marking enhances male reproductive success when females can choose their mates (Thonhauser et al., 2013). The scent of male urine is attractive to females and exposure to male urine influences female behavior and physiology by accelerating puberty, synchronizing estrus, and inducing vaginal opening (Beynon and Hurst, 2003; Stopka et al., 2007, 2012; Stopková et al., 2009, 2014; Jouhanneau and Keller, 2013; Mucignat-Caretta and Caretta, 2014; Wyatt, 2014). The effects of male urinary odor on females are influenced by male MUPs or their volatile ligands (Jemioło et al., 1985; Harvey et al., 1989; Novotny et al., 1990; Jemioło et al., 1991). Thus, scent marks and sexual pheromones are secondary sexual traits, analogous to the colorful and conspicuous displays of peacocks (Penn and Potts, 1998; Zala et al., 2004; Thonhauser et al., 2013).

### MUP Genes

**Figure 2:**
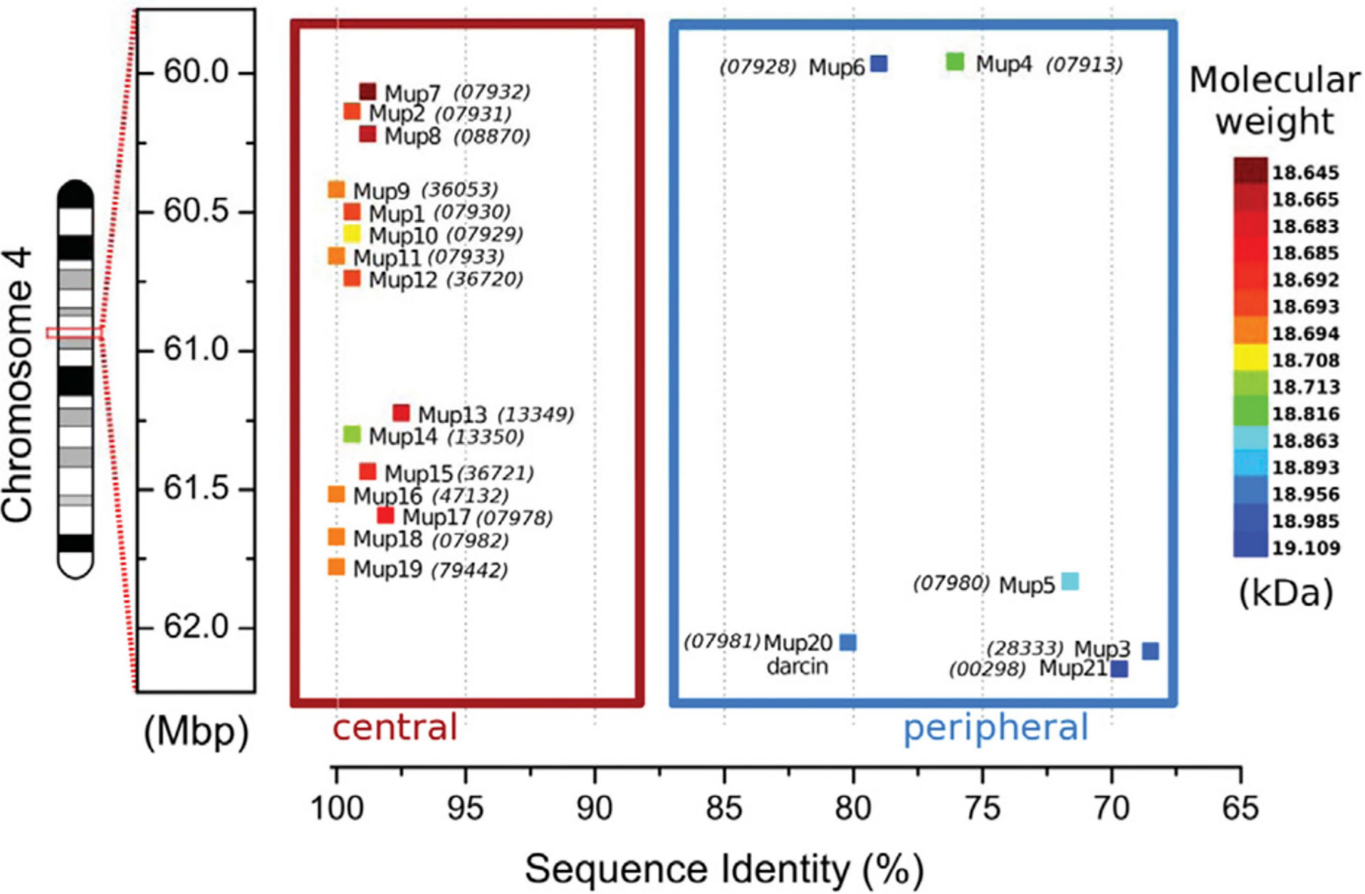
MUP Gene Cluster of House Mice. MUP genes, indicated by colored squares, are closely linked in a large (2 Mb) region on chromosome 4 that contains up to 21 tandemly coding MUP genes and *ca*. 30 non-coding pseudogenes (Logan et al., 2008; Mudge et al., 2008; Phelan et al., 2014). MUP loci have been classified depending on their location inside of this cluster: the six Class A or “peripheral” MUPs (*Mup3, 4, 5, 6, 20*, and *21*, in the blue box) share *ca*. 88% similarity (“<82% mature protein sequence identity”), and these MUPs are likely more ancestral. The 15 Class B or “central MUPs” (in the red box) are nearly identical and show >97% mature protein sequence similarity and some differ by a single amino acid substitution. These highly homologous MUPs are likely recent duplications (Logan et al., 2008). Class A and B MUPs do not appear to differ in their gene expression levels in the liver (see Figure 4 below). The coloration of the MUP gene corresponds to the molecular weight of the mature protein upon translation. The sequence identity percentage is relative to the most common mature amino acid sequence that is shared by genes *Mup9*, *11*, *16*, *18*, and *19*. Numbers in parentheses correspond to the MGI protein identification number. All numbers are prefixed with “OTTMUSP000000.” Figure used with permission from Phelan et al. (2014).

House mice have *circa* 21 functional MUP genes and *ca*. 30 non-coding pseudogenes closely linked in a large cluster (Logan et al., 2008; Mudge et al., 2008; Charkoftaki et al., 2019; note that *Mup* is italicized whenever referring to a specific genetic locus or transcript, for example, *Mup1*; Figure 2). MUP genes are found in most placental mammals, though most species have only a single gene. Humans have one MUP gene, but it is dysfunctional and we are the only placental mammal lacking any active MUPs. MUPs likely evolved from another group of lipocalins, called odorant-binding proteins (OBPs; Charkoftaki et al., 2019; but see Igarashi et al., 1992). MUP genes are highly homologous and targeted methods, such as qPCR, do not necessarily amplify only one specific MUP locus (Holloway et al., 2006; Thoß et al., 2016). Only one study to our knowledge has measured genetic variation of MUPs within populations of wild house mice, and contrary to what is often suggested, MUPs have unusually *low* rather than high levels of individual variation (Thoß et al., 2016). MUPs show differences in expression across loci (Shi et al., 1989), but, contrary to what is often assumed, they do not show constitutive gene expression; as we show below, transcription is dynamically regulated and different MUPs are regulated in a different manner (Connerney et al., 2017).

Some MUPs are expressed in saliva, tears, vaginal and other glandular secretions (Shaw et al., 1983; Shahan et al., 1987; Stopka et al., 2016; Černá et al., 2017; Stopková et al., 2017, 2021), as mentioned above. For example, *Mup4* is expressed in glands near the nasal cavity and in the nasal mucosa and the vomeronasal organ, where its proteins are suspected to transport ligands to olfactory receptors (Miyawaki et al., 1994; Cavaggioni and Tirindelli, 1999; Utsumi et al., 1999; Sharrow et al., 2002; Stopková et al., 2016). Thus, the original label “Major Urinary Proteins” turned out to be misleading because MUP proteins are not expressed only urine, which is also why the term “MUP protein” is used and not as redundant as it might seem.

### MUP Proteins

MUPs are mainly synthesized in the liver (Finlayson et al., 1965), and they are among the most highly expressed genes in the liver: *ca*. 5% of the total hepatic mRNA in adult male mice consists of MUPs (Knopf et al., 1983). MUP are then released into the serum, filtered by the kidney, and excreted in the urine (Flower, 1996; Åkerstrom et al., 2000; Flower et al., 2000). It has long been known that male mice excrete high levels of protein in their urine (Parfentjev, 1932; Parfentjev and Perlzweig, 1933), and their urinary proteins are mainly composed of MUPs (Finlayson et al., 1963). It is often stated that around 95– 99% of urinary protein consist of MUPs (Humphries et al., 1999; Hurst and Beynon, 2004), but these may be overestimates. MUP-derived peptides accounted for 85% of the total urinary protein (from high-resolution mass spectrophotometry) of on wild-derived *M. musculus musculus* (Enk et al., 2016). Contrary to what was long assumed, gel-based methods do not separate different MUP proteoforms (Thoß et al., 2016), and quantifying the abundance of different proteoforms remains a challenge for proteomic methods (Enk et al., 2016).

MUPs undergo post-translational modifications in which a carbohydrate is attached (glycosylation), and there must be extensive modifications for these 162 amino acid proteins to expand to a mature protein 40 kDa in size. MUP15 has been shown to be glycosylated (Clark et al., 1985) and the resulting glycoprotein has a higher mass and exhibits a highly heterogeneous glycosylation pattern (Mechref et al., 2000). The relative ratio of protein masses predicted from mRNA generally matches the observed ratios of masses in protein data, suggesting that post-transcriptional modifications do not influence estimates of variation (Sheehan et al., 2016). Yet, MUP3 (referred to as “B6 gene18” Mudge et al., 2008), which is also glycosylated, does not show up on standard analyses of urine protein content using mass spectrometry due to the change in its mass, even though it is detectable using other methods. Gel electrophoresis shows MUP expression in the urine of B6 males but not females and that lack of transcription analyses have probably misinterpreted expression patterns in wild populations (Sheehan et al., 2019). The effects of glycosylation on the functions of MUPs and their expression in different tissues are not understood and deserve more attention.

The cavity of each MUP20 protein has 14 amino acids associated with ligand binding, and a single amino acid substitution can alter ligand binding affinity and specificity (Ricatti et al., 2019). Yet, very few amino acid substitutions are found in the interior hydrophobic binding cavity of MUPs, as most occur on the protein surface (Darwish et al., 2001; Beynon et al., 2002; Sheehan et al., 2019). Surface substitutions do not likely influence ligand binding affinity, though they might alter the shape of the binding cavity (Darwish et al., 2001; Beynon et al., 2002), and variation in surface-exposed residues might influence detection by V2R receptors in the vomeronasal organ (Chamero et al., 2007, 2011; Phelan et al., 2014; Sheehan et al., 2019). Amino acid variations may also affect the stability of different MUP proteoforms, for example, MUP20 has been found to be more stable at higher concentrations of a denaturing agent (urea) compared to a central proteoform (MUP11; Phelan et al., 2014).

Levels of urinary protein output show differences between wild house mice versus laboratory mice, and between wild mice kept in standard cages versus seminatural conditions (Enk et al., 2016; Thoß et al., 2019; Luzynski et al., 2021). These results indicate that it is crucial to study MUP gene and protein expression in wild mice and preferably living in natural or naturalistic social conditions to understand their functions, as we show next in more detail.

### Sexually Dimorphic MUP Expression

**Figure 3:**
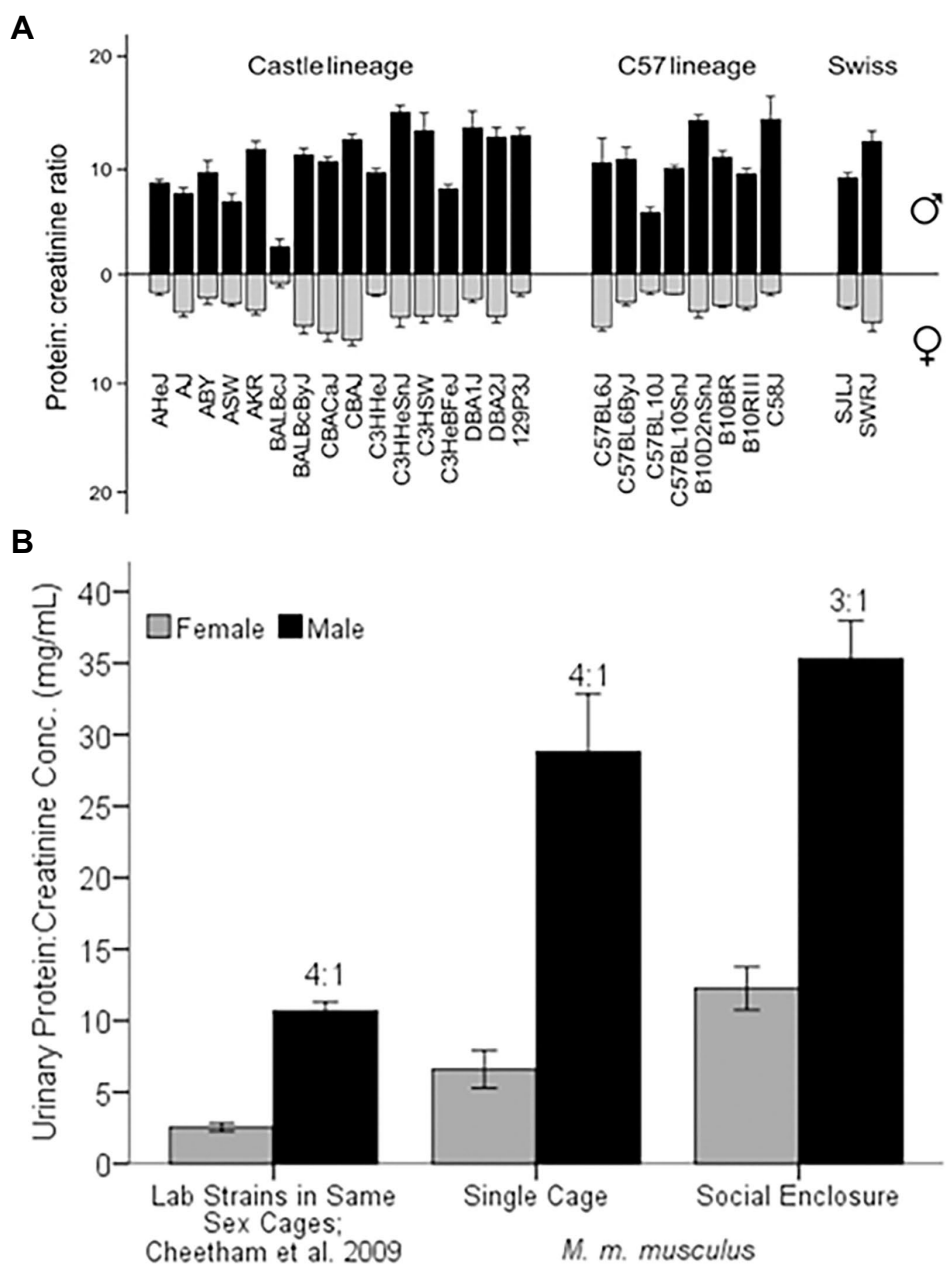
Sex Differences in Urinary Protein Excretion. **(A)** Comparison of 26 strains of laboratory mice (protein:creatinine concentration in mg/ml) for males and females, represented as black and gray bars, respectively. Figure used with permission from Cheetham et al. (2009). **(B)** MUP excretion of laboratory versus wild-derived house mice. Laboratory strains were housed in same-sex cages of 5 individuals of the same strain (Cheetham et al., 2009). Wild-derived *M. musculus musculus* were either singly housed (Single Cage) or living in seminatural conditions (Social Enclosure) at 1:1 sex ratio (modified from Luzynski et al., 2021). The ratio above the black bars is the male:female ratio of protein excretion. Error bars indicate ±1 SEM.

It has long been known that male laboratory mice excrete more protein in their urine (Wicks, 1941) and synthesize more MUP mRNA (Sampsell and Held, 1985) than females. MUP urinary excretion begins at puberty (Wicks, 1941; Thoß et al., 2015), and numerous studies have documented male-biased MUP expression in mice, though these estimates vary considerably. In laboratory mice, males express between 2 to 8 times more urinary protein (Stopka et al., 2007; Mudge et al., 2008; Cheetham et al., 2009; Novikov et al., 2009), and 5- to 10-fold more MUP mRNA in the liver than females (Szoka and Paigen, 1978; Hastie et al., 1979; Derman, 1981). The amount of protein excreted and the degree of sexual dimorphism varies among laboratory strains (Cheetham et al., 2009; Figure 3A). One strain, BALB/cJ, has unusually low levels of urinary protein excretion due to a mutation in a regulatory gene (Jiang et al., 2017; see more on gene regulation below).

Wild-derived house mice also show male-biased urinary protein excretion when housed in standard cages and in seminatural conditions (Figure 3B; Stopková et al., 2007; Thoß et al., 2019; Luzynski et al., 2021). Wild-derived *M. musculus musculus* show a 4:1 male-biased urinary protein excretion in the laboratory (Luzynski et al., 2021), which is equivalent to the grand mean sex bias found in laboratory strains, despite that wild-derived males excrete nearly three times more protein. *M. musculus musculus* males produce more urinary protein than *M. musculus domesticus* (Stopková et al., 2007; Hurst et al., 2017; see more below), though both European subspecies show a 3.5 to 4 fold male bias in urinary protein excretion in the laboratory. Thus, both sexes show higher mean protein output in seminatural social contexts and male-biased excretion is somewhat less pronounced than in laboratory conditions (3:1 versus 4:1 respectively; Thoß et al., 2019; Luzynski et al., 2021).

**Figure 4:**
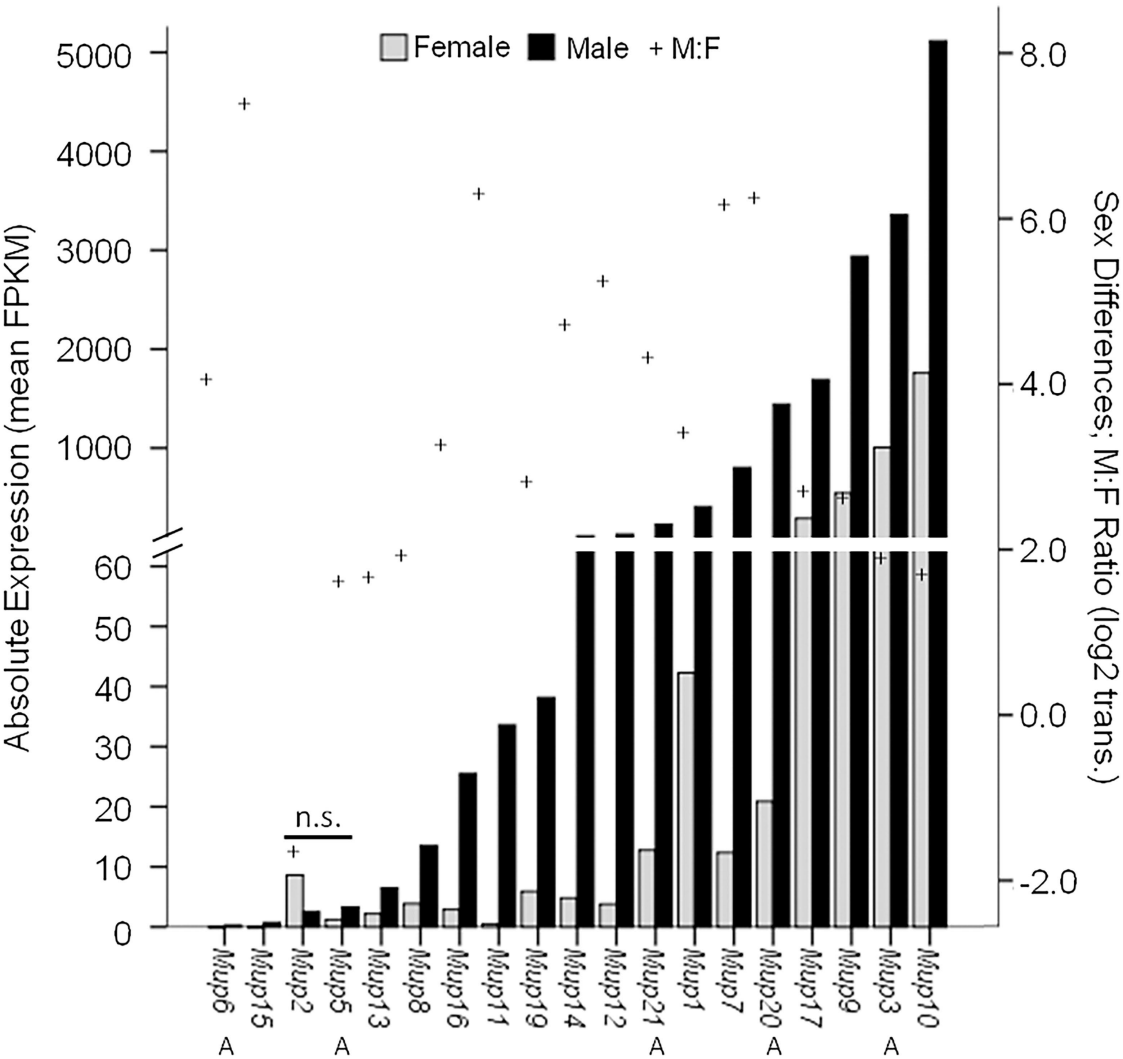
Sex Differential MUP Gene Expression. MUP expression of male (black bars; *n* = 18) and female (gray bars; *n* = 5–9) was measured in normalized FPKM units (fragments per kb transcript per million mapped fragments) and MUP transcripts are shown in order of absolute expression levels of males; Swiss CD-1 mice, ICR strain. Our estimate of sex differential gene expression [log2(Male:Female); +] and Class A (peripheral) MUPs (A) are also indicated; others are Class B or central MUPs. Data from Connerney et al. (2017, Supplementary Table S2).

Some studies suggest that certain MUPs, such as *Mup7*, *11*, *20*, and *21*, show particularly high levels of expression in males and little if any in females (Norstedt and Palmiter, 1984; Hurst et al., 2017). However, because targeted methods, such as qPCR, do not necessarily discriminate different MUPs due to their high homology, it is usually unclear which MUP or MUPs are measured (e.g., see Holloway et al., 2006). Only one study to our knowledge has used RNA sequencing (RNA-seq), a more precise method for comparing MUPs (see Supplementary Table S2 in Connerney et al., 2017), and we plotted these results (Figure 4). Most MUP transcripts (17/19) showed significant male-biased expression (26-fold sex difference on average); however, there was enormous variation in the degree of sex-biased expression across loci (from 0- to 150-fold by our estimate). The most sexually dimorphic MUPs were *Mup7, 20, 11, 15*; only *Mup2* and *5* showed no significant sex differences. Males showed much variation in absolute expression levels across loci, as some MUPs had very high (*Mup7, 20, 17, 9, 3, 10*), whereas others had low expression (*Mup6, 15, 2, 5, 13*), comparable to females. Females had low expression for most MUPs, but also showed variation across loci and some (*Mup17, 9, 3, 10*) had higher levels than most MUPs in males. Only *Mup7* and *20* showed both large sex differences and high levels of male expression. We noticed that the same MUPs had either low (*Mup6, 15, 2, 5, 13*) or high expression (*Mup17, 9, 3* and *10*) in both sexes, and we found a correlation between male and female expression across loci (*r* = 0.87; *p* = 2.6 × 10^−6^; df = 18; not shown).

The results of this study show that most MUPs have sexually dimorphic expression, but that there are large differences in expression across MUP loci in both sexes and especially in males. The expression of different MUPs is correlated between the sexes, suggesting similarities as well as differences in their regulatory mechanisms. Since there is so much variation in expression across MUP loci, results from studies measuring the expression of a specific MUP are not likely to generalize to other loci and therefore should not be extrapolated (especially if the targeted method is specific). This RNA-seq study was conducted on one strain of domesticated mice in the laboratory, and therefore, more such studies are needed on wild mice living in more natural social contexts. The *main results* from this study reinforce the importance of this caveat: the expression of MUPs also showed dramatic changes following endocrine manipulation (Supplementary Table S2 in Connerney et al., 2017; see more below). Before addressing hormonal mechanisms that control MUP gene regulation, including sex differences, we examine how MUP expression is dynamically regulated by several factors that can magnify or abolish sex differences.

## Factors That Influence the Regulation of MUP Expression

The amount and types of MUPs that mice excrete are regulated depending upon a variety of factors, including age, social interactions, social status, and health, and males and females show similarities and differences in how they regulate MUP expression (Table 1). For example, males upregulate MUP expression during puberty and after acquiring dominant social status, whereas only females regulate expression depending upon estrous cycle. It is premature to make general conclusions about sex differences in MUP regulation, however, because few studies have simultaneously compared males and females, and there are fewer studies on females than males. For example, male MUP expression is downregulated due to fasting (dietary restriction), infection, immune activation, microbiota depletion, and old age, and thus MUP output is a condition-dependent trait. Poor health or condition feminizes male MUP expression and can abolish sex differences. For most of these factors, however, it is still unclear whether females show similar condition dependence. Some factors, such as social status, infection, and immune activation, have been shown to result in perceptible changes in odor as well as MUP expression, though others have not yet been tested. The ecological relevence these findings are still unclear, as most studies come from the laboratory, and as mentioned above, mice alter how they regulate MUP expression in natural social contexts, as we examine next in more detail.

**Table 1:** Factors affecting MUP expression.

Factor	Male regulation	Female regulation
Sexual maturity	↑ after puberty (Payne et al., 2001; Thoß et al., 2015)	Consistent expression from ages 20 to 100 days (Payne et al., 2001)
Housing (standard laboratory cages vs. social conditions)	↑ in seminatural conditions versus standard cages (Nelson et al., 2015; Enk et al., 2016; Lee et al., 2017; Thoß et al., 2019; Luzynski et al., 2021) Solitary > group housed (6 per cage) at age 60 days (Mucignat-Caretta et al., 2014)	↑ in seminatural conditions versus standard cages (Stockley et al., 2013; Thoß et al., 2019; Luzynski et al., 2021)
Social status	↑ in territorial dominants but not subordinates (Nelson et al., 2015; Lee et al., 2017; Thoß et al., 2019; Luzynski et al., 2021)	No change (Thoß et al., 2019; Luzynski et al., 2021)
Intermittent or indirect agonistic and other social interactions	↑ in winners (“social dominants”) in dyadic interactions (Guo et al., 2015; Lee et al., 2017) ↑ with territory defense (Garratt et al., 2012) ↑ with intersexual indirect contact and ↓ with intrasexual indirect contact (Janotová and Stopka, 2011)	↑ with intersexual indirect dyadic interactions (Stopka et al., 2007; Janotová and Stopka, 2011) ↑ with territory defense (Garratt, et al., 2011b) ↑ with aggressive behaviors (Stockley et al., 2013)
Estrous stage	N/A	↑ with estrus onset (Stopka et al., 2007)
Dietary restriction	↓ with dietary restriction (Hui et al., 2009; Giller et al., 2013; Mitchell et al., 2015)	↓ *Mup1* transcription with dietary restriction (Van Schothorst et al., 2006)
Cold stress	↑ with housing at 4°C ambient temperature (Liu et al., 2019)	No reports
Health (infection and immune activation)	↓ with infection and immune activation (Glibetic and Baumann, 1986; Isseroff et al., 1986; Gervois et al., 2004; Litvinova et al., 2005; Manivannan et al., 2010; Lopes and König, 2016; Deslyper et al., 2019; Ware et al., 2019; Oldstone et al., 2021; but see Lanuza et al., 2014) ↓ transcription with immune activation (Glibetic and Baumann, 1986; Gervois et al., 2004; Lopes and König, 2016)	↓ with infection (Isseroff et al., 1986; Deslyper et al., 2019)
Microbiota depletion	↓ transcription in germ-free mice (Weger et al., 2019)	↓ transcription in germ-free mice (Weger et al., 2019)
Toxin exposure	↑ with iron overloaded diet (Petrak et al., 2007)	No reports
Aging	↓ in old, senesced males (c. 26 mo) vs. middle-aged males (c.14 mo; (Garratt, et al., 2011a))	No reports

*MUP gene expression in the liver and protein in the urine is regulated depending upon several factors, which can magnify or abolish sexual dimorphisms. Note that results based on targeted methods for measuring expression do not necessarily generalize to all loci*.

### Social Status

**Figure 5:**
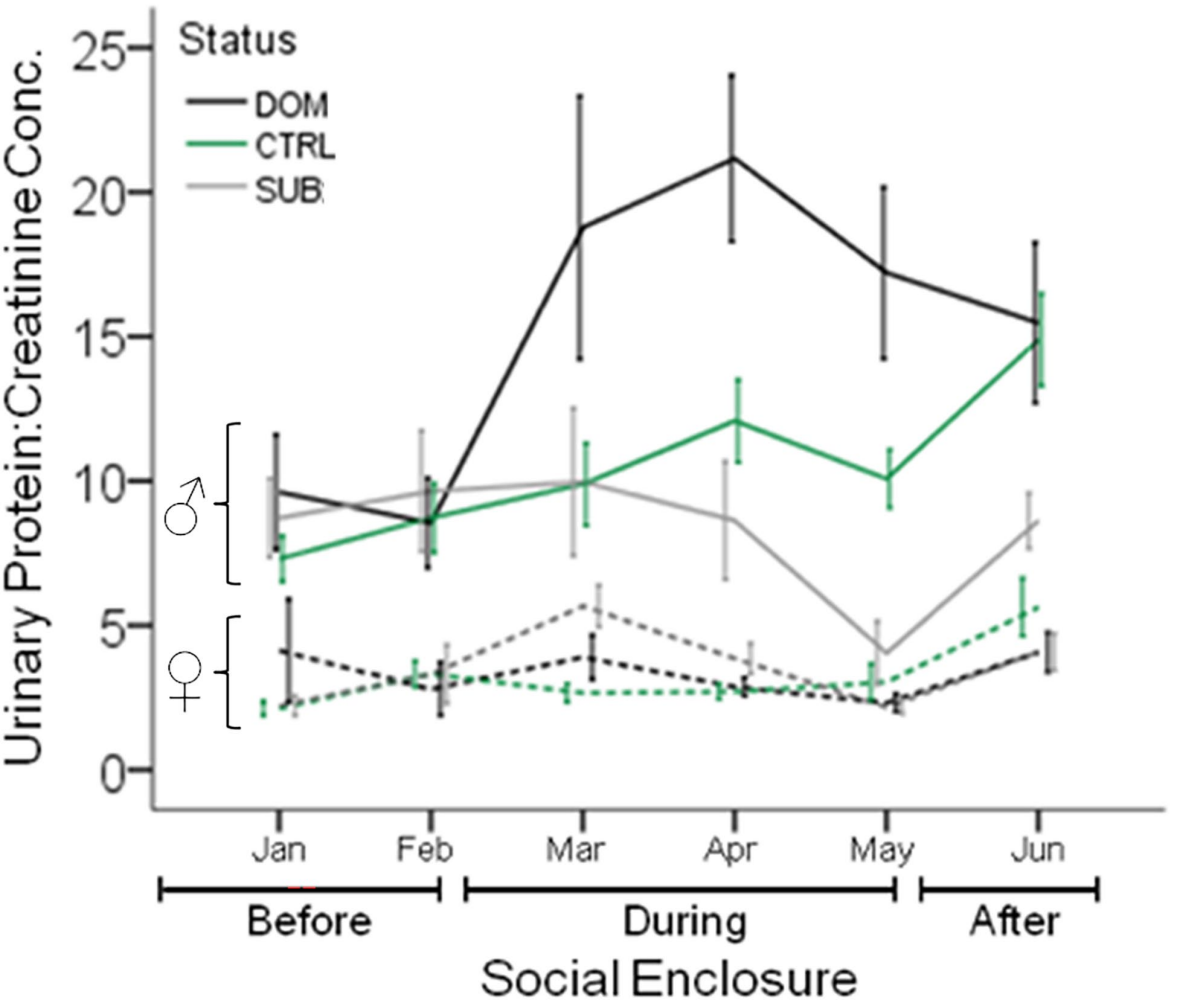
Urinary Protein Excretion in Seminatural Contexts. The urinary protein excretion of wild-derived male and female house mice over time, before, during, and after their release into seminatural conditions. Lines show the mean protein excretion of males (solid lines) and females (dashed lines) caged singly in the laboratory (Before), and also while they were living in large enclosures for 12wk (During), and for 4 weeks after capture and being returned to single-caged housing (After). Dominant (DOM) versus subordinate (SUB) status is indicated by black and gray lines, respectively. Control mice (CTRL) are indicated by the green line. Error bars display ±1 SEM. Data from Thoß et al. (2019).

After releasing wild-derived house mice kept in the laboratory conditions, males significantly increase their urinary protein excretion once they acquire a territory and become socially dominant in seminatural conditions (Thoß et al., 2019; Luzynski et al., 2021; Figure 5). Subordinate males, which do not acquire a territory, do not show a change in urinary protein excretion over time or compared to controls kept in the laboratory during the same time (nor do they differentially downregulate specific MUP proteoforms; Thoß et al., 2019). Dominant males also excrete higher levels of several MUPs, including MUP2, 5, 17, and 20, in their urine compared to subordinates (Thoß et al., 2019). Interestingly, males upregulate the expression of some MUPs but downregulate others after being released into naturalistic social conditions (Enk et al., 2016). Protein excretion in the laboratory does not predict male social status in seminatural enclosures, and the differences in MUP excretion between dominant versus subordinate males in the enclosures are diminished after returning males to their cages. In contrast, females do not adjust their protein excretion depending upon their social status. Sociality has been observed to correlate with increased protein excretion in females (Garratt et al., 2011b; Stockley et al., 2013), and yet wild mice show lower sexual dimorphism in social contexts compared to the same mice in the laboratory (singly housed in cages; Figure 3B; Thoß et al., 2019; Luzynski et al., 2021). These findings indicate that, in addition to urinary protein excretion being male-biased, there are also sex differences in how mice regulate MUP excretion according to social status. MUPs are not only sexually dimorphic; in more natural conditions they also show a male dimorphism, like the secondary sexual traits of some other species (Számadó and Penn, 2018).

### Agonistic Interactions

Studies of *M. musculus* and *M. laboratorius* indicate that MUP excretion depends on male aggression and competitive ability (Guo et al., 2015; Nelson et al., 2015; Lee et al., 2017; Thoß et al., 2019; Luzynski et al., 2021). Males that experienced repeated social defeat do not downregulate MUP excretion, but remained consistent with pre-interaction levels; any increase was to a lesser degree compared to dominant individuals (Nelson et al., 2015; Lee et al., 2017; Thoß et al., 2019). Two studies found that MUP excretion predicted subsequent aggression (Janotová and Stopka, 2011) or territorial dominance (Nelson et al., 2015), whereas other studies did not observe this pattern (Guo et al., 2015; Lee et al., 2017; Thoß et al., 2019; Luzynski et al., 2021). Male competitive ability is associated with the regulation of specific MUP isoforms: winners and socially dominant males upregulated MUP20 (Guo et al., 2015; Nelson et al., 2015; Lee et al., 2017; Thoß et al., 2019), as well as MUP2, MUP5, and MUP17 (Thoß et al., 2019). A downregulation of hepatic *Mup20* gene expression and MUP20 in urine has been observed in subordinate C57BL/6 males (Guo et al., 2015). Social hierarchies form within a few days (Guo et al., 2015; Lee et al., 2017) and are relatively stable in seminatural conditions (Thoß et al., 2019; Luzynski et al., 2021). However, MUP excretion changes in the laboratory depending upon social conditions or density, as evidenced by a decrease in MUP excretion by males upon moving from paired-housing to group-housing (Lee et al., 2017), and upon removal from social conditions to single-housing (Thoß et al., 2019). These studies are consistent with the hypothesis that MUP output is regulated depending upon social status. These findings suggest that the volatile ligands transported by urinary MUPs are important for signaling social status and that the persistence of male pheromones in scent marks should be prolonged by the high protein excretion of dominant males.

Thus, MUP excretion is more complex and dynamic in social contexts than in the laboratory, which raises caveats for studies conducted in the laboratory and also for surveys of wild house mice. After being trapped and housed in cages, dominant males reduced hepatic MUP20 protein expression by day 14 and urinary protein excretion by day 28 (Garratt et al., 2011b; Luzynski et al., 2021). Variation in the length of time that mice are kept in captivity can therefore affect MUP expression, and especially when animals are caged for more than 28 days (Thoß et al., 2019). To our knowledge, no studies on wild-caught mice have controlled for male social status or the amount of time in captivity. In the next section, we examine the complex mechanisms controlling the regulation of MUP expression in house mice.

## Molecular Mechanisms Controlling Adult MUP Gene Transcription

The proximate mechanisms controlling sexually dimorphic MUP excretion are being worked out in remarkable detail and they are surprisingly complex, even in laboratory mice. These mechanisms are intensively studied because MUPs are used as a model protein for investigating sex differences in hepatic gene expression in physiology and biomedical sciences. These studies show how the pituitary gland regulates MUP gene expression by releasing endocrine signals that trigger a complex cascade of biochemical changes in the liver.

### Endocrine Mechanisms

Studies on the MUPs of laboratory rats (originally labeled *α2_u_-globulins*) showed that sex differences in MUP expression are controlled by growth hormone (GH) and thyroxine [T_4_; see references cited in (Knopf et al., 1983)]. Subsequent studies on laboratory mice showed that hepatic MUP synthesis is similarly under multihormonal control, involving testosterone (T), GH, and T_4_ (Knopf et al., 1983). Surgical removal of the pituitary gland (hypophysectomy) makes it possible to examine the effects of pituitary hormones. Hypophysectomized female mice and GH-deficient *little* mice and have reduced MUP RNA expression in the liver, and administering either GH or T_4_ increased MUP production in these mice. T had relatively little effect on the MUP RNA levels in hypophysectomized females, although it increased MUP production in normal females. GH and T_4_ had the most pronounced effects on liver MUP RNA of hypophysectomized mice when administered together (even above normal physiological ranges). T, T_4_, and GH appear to differentially regulate the expression of distinct MUPs (Knopf et al., 1983; Kuhn et al., 1984). A study using RNA-seq, mentioned above, found that hypophysectomy reduced gene expression of MUPs in both sexes (6-fold drop on average), and found variation in regulation across MUP transcripts (Connerney et al., 2017).

Many studies have shown that it is not the amount of circulating GH, but rather *the pattern of its pituitary release* that explains baseline sex differences in MUP expression. Here, we focus on GH- and T-mediated MUP expression, the best understood mechanisms, though additional regulatory mechanisms have been found that deserve more attention. For example, prolactin (also secreted by the pituitary gland) triggers milk production upon pregnancy, inhibits the expression of male-predominantly expressed hepatic genes, including *Mup1*, and upregulates mRNA expression of female-predominant genes (Sato et al., 2017). This finding could explain the increased MUP output of females in seminatural conditions (Stockley et al., 2013; Thoß et al., 2019; Luzynski et al., 2021). Prolactin did not reduce the levels of serum *levels* of GH and T in males (Sato et al., 2017), but its effects were not necessarily independent of hormones, contrary to what was suggested, because changes in the pulsatile secretion of these hormones were not investigated.

### Growth Hormone

The effects of GH on growth and metabolism are well known, though interestingly, it is the *pulsatile* GH secretion in the pituitary that is necessary for normal postnatal growth, and especially accelerated growth during the peri-pubertal period. This peptide hormone has many pleiotropic effects as it affects reproduction, as well as growth, even though it is not usually considered to be a sex hormone. GH plays a key role in regulating MUP gene transcription and sex differences in the expression of MUPs and many other genes in the liver.

Studies on rats and mice have shown that the pattern of pituitary GH secretion is the key regulator of sex differences in the expression of MUPs and other genes in the liver (Mode et al., 1982; Norstedt and Palmiter, 1984; McIntosh and Bishop, 1989; Waxman and O’Connor, 2006; Zhang et al., 2012). Males have a highly pulsatile release of GH, whereas GH secretion in females is nearly continuous (Tannenbaum and Martin, 1976; MacLeod et al., 1991; Painson et al., 1992). In male mice, GH ultradian rhythms (rhythms that occur within a 24 h interval) exhibit regular periodicity with peak secretion periods occurring soon after the start of the light phase (2.5 h after lights on; Steyn et al., 2011). Males release *ca*. 5 secretory GH bursts per hour, and these multicomponent peaks last *ca*. 2 h and have an amplitude of *ca*. 200 ng/ml. The liver is the most sensitive target tissue for GH, and pulsatile release of GH release generates a male pattern in MUP gene expression and hundreds of other genes in the liver, which control metabolism of steroids, lipids, and toxins (Mode et al., 1982; Norstedt and Palmiter, 1984; Macleod and Shapiro, 1989; MacLeod et al., 1991; du Sert et al., 2020). Studies in rats found that it is the long inter-pulse interval with low plasma GH levels, rather than changes in pulse amplitude, duration, or frequency, that generates male versus female hepatic gene expression profiles (Pampori et al., 1991; Le Tissier et al., 2018). Pulsatile GH secretion is difficult to study in mice, though an alternative method has been developed for mice, which confirmed that plasma GH concentration patterns in mice are similar to other mammals (Xu et al., 2011). Additionally, experiments using continuous GH (cGH) infusion, which generate a female-like GH pattern, also suppress MUP output of male mice, and conversely intermittent GH administration results in male levels of MUP output in females (Gustafsson et al., 1983; Norstedt and Palmiter, 1984; Al-Shawi et al., 1992; Johnson et al., 1995; Metcalf et al., 2000). Infusing males with continuous GH repressed 86% of male-biased genes and induced 68% of female-biased genes within 4 days of infusion (Lau-Corona et al., 2017). This method of manipulating GH secretion has helped to unravel the molecular mechanisms through which GH regulates MUP gene transcription.

### GH Regulation of MUP Gene Transcription

**Figure 6:**
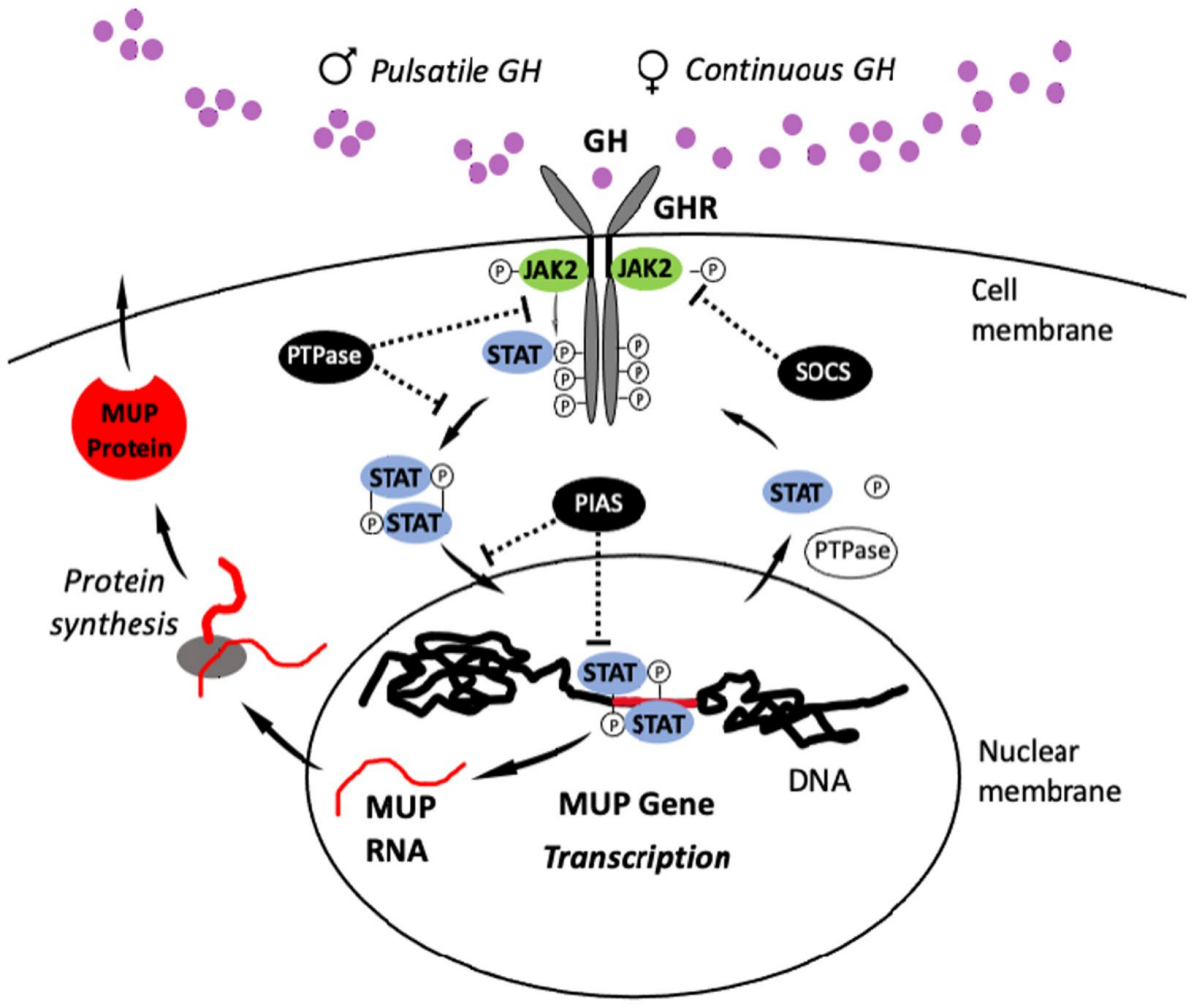
JAK2-STAT5 Signaling Pathway. The pulsatile pattern of GH release in male mice activates the JAK2-STAT5 signaling pathway (or “signaling cascade”): Circulating GH binds to transmembrane GH receptors (GHR), which are members of the cytokine receptor family and widely expressed on the surface of target cells in the liver. GH binding induces a conformational change in GHRs that activate Janus kinase 2 (JAK2), and then this enzyme phosphorylates the cytoplasmic domain of the GHR, generating docking sites for the transcription factor STAT5b. After STAT5b binds to these sites, it undergoes JAK2-catalyzed tyrosine phosphorylation and dissociates from the GHR to form homodimers (e.g., STAT5-STAT5) or heterodimers with other STAT proteins. STAT5 dimers enter the nucleus and bind response elements in gene regulatory regions, initiating transcription (Udy et al., 1997; Teglund et al., 1998). After transcription, STAT5b is deactivated by phosphotyrosine phosphatase (PTPase). STAT5b can then enter into another cycle of JAK2-catalyzed phosphorylation, and it can undergo multiple rounds of this cycle in response to a single male GH pulse (Gebert et al., 1999). In response to female-like patterns of GH release, STAT5b cycles are terminated more rapidly through STAT5b inhibitors, including PTPase, PIAS (protein inhibitors of activated STATs), and SOCS (suppressor of cytokine signaling)/CIS. MUP gene expression is repressed in females by CUX2, a small interfering RNA (siRNA; Conforto et al., 2012). Figure adapted from Waxman and O’Connor (2006).

GH pituitary secretion controls sex differences in hepatic MUP gene expression through the JAK2-STAT5 signaling pathway in target cells (Figure 6; Holloway et al., 2008). This signaling pathway is an example of signal transduction, that is, the conversion of one type of signal to another type. It begins with GH binding to GH receptors (GHR) on target cells in the liver, which activates a key transcription factor, STAT5 (signal transducer and activator of transcription), which then enters the nucleus and initiates MUP gene transcription. Activation of STAT5 triggers a biochemical cascade of reactions (signaling cascade), so that the effects of a few GH molecules can be amplified through positive feedback to induce transcription of large numbers of MUPs (and other sex-biased genes), and the effects of GH can be dampened through negative feedback loops.

GH pulse rate regulates the expression of MUP genes *via* the JAK2-STAT5 pathway, and approximately 1,000 other genes in the liver (Udy et al., 1997; Teglund et al., 1998). The deletion of one or both Stat5a and Stat5b genes dramatically reduces male MUP gene expression levels (Udy et al., 1997). The deletion of Stat5a has no effect on female MUP levels, whereas deleting Stat5b reduces MUP gene expression, and the deletion of both Stat5a and Stat5b abolishes MUP protein synthesis (Teglund et al., 1998). STAT5-knockout (KO) mice have reduced hepatic MUP expression, especially on males (Clodfelter et al., 2006; Holloway et al., 2006). STAT5b appears to inhibit expression MUP expression in females (Waxman and O’Connor, 2006). Continuous GH infusion (cGH) overrides the normal pulsatile GH pattern of males and abolishes male-specific, pulsatile pattern of hepatic STAT5 activity (Zhang et al., 2012). cGH downregulates MUPs and other male-biased genes, whereas it upregulates female-biased genes in the liver (Holloway et al., 2006). There is much variation in how expression is regulated across MUP loci in response to changes in GH pulses, as mentioned above, and GH pulse rates regulate the expression of some MUPs (*Mup1, 2, 6*, and *8*) more rapidly than others (Waxman and O’Connor, 2006; Connerney et al., 2017).

There are other transcription factors that regulate MUP gene expression, such as the regulatory protein zinc fingers and homeoboxes 2 (Zhx2). Protein excretion varies among strains of laboratory mice, and BALB/cJ mice have the lowest protein output (Figure 3). Their low protein excretion is due to a mutation caused by the insertion of an endogenous retroviral element into the Zhx2 promotor on chromosome 15 (Jiang et al., 2017). Zhx2 suppresses expression of other genes in the liver, whereas it promotes the expression of a number of MUP genes by binding and activating promotors (Jiang et al., 2017). Zhx2 is necessary for the high levels of hepatic MUP expression of males, and several MUP genes (*Mup20, Mup3*, and class B *Mup7, Mup10*, and *Mup19*) show differential responsiveness to Zhx2. It is not known whether Zhx2 influences normal physiological variations of MUP output between or within the sexes. These findings raise the question: how do the transcription factors, STAT5 and Zhx2, initiate transcription in the nucleus?

### GH Regulates Chromatin Accessibility

Pulsatile GH release controls transcriptional regulation of MUPs and other male-biased genes by dynamically regulating chromatin accessibility, histone modification, and binding of transcription factors (Connerney et al., 2017; Lau-Corona et al., 2017). Chromatin in genomic regions that are transcriptionally active loses its condensed structure and DNA is exposed. These open sites are sensitive to cleavage by DNase I and DNase I hypersensitive sites (DHSs), which are used as markers for active regulatory regions. DHSs contain key regulatory elements, including enhancers, promoters, insulators, and silencers, and they are often flanked by histone modifications. Experimental cGH closes many male-biased DHSs in the liver of male mice and opens female-biased DHSs (Ling et al., 2010). Mapping DHSs has revealed that sex differences in chromatin accessibility are associated with sex differences in gene expression (Ling et al., 2010; Sugathan and Waxman, 2013). Sex-biased STAT5 chromatin binding is enriched at sex-biased DHSs and positively correlated with sex-biased activating histone marks and negatively correlated with repressive marks (Zhang et al., 2012; Sugathan and Waxman, 2013). These studies show how the endogenous rhythms of male GH pulsatile release open and then close chromatin at regulatory sites of MUPs and other sexually dimorphic genes in association with temporal changes in transcriptional activation (Connerney et al., 2017). A cGH infusion study examined global gene expression in the liver over time to determine the transcriptional events that result in the feminization of MUPs and other male-biased genes (Melia and Waxman, 2019). As expected cGH infusion induced male-biased gene repression and female-biased gene derepression, and these changes occurred in distinct waves over time. These waves of transcription were initiated by a hierarchical transcriptional network involving several sex-biased transcription factors. More recently, sex-dependent binding of STAT5 to chromatin has been shown to be closely linked to the sex-dependent demethylation of distal regulatory elements that map to genes that show sex-biased expression (Hao and Waxman, 2021).

Thus, GH-mediated regulation of MUP gene expression involves complex interactions between transcriptional networks, genomic regulatory elements, and epigenetics. Given its key role in controlling MUP expression, we next examine how GH secretion is regulated in adult mice.

### Regulation of Pulsatile GH Secretion

**Figure 7:**
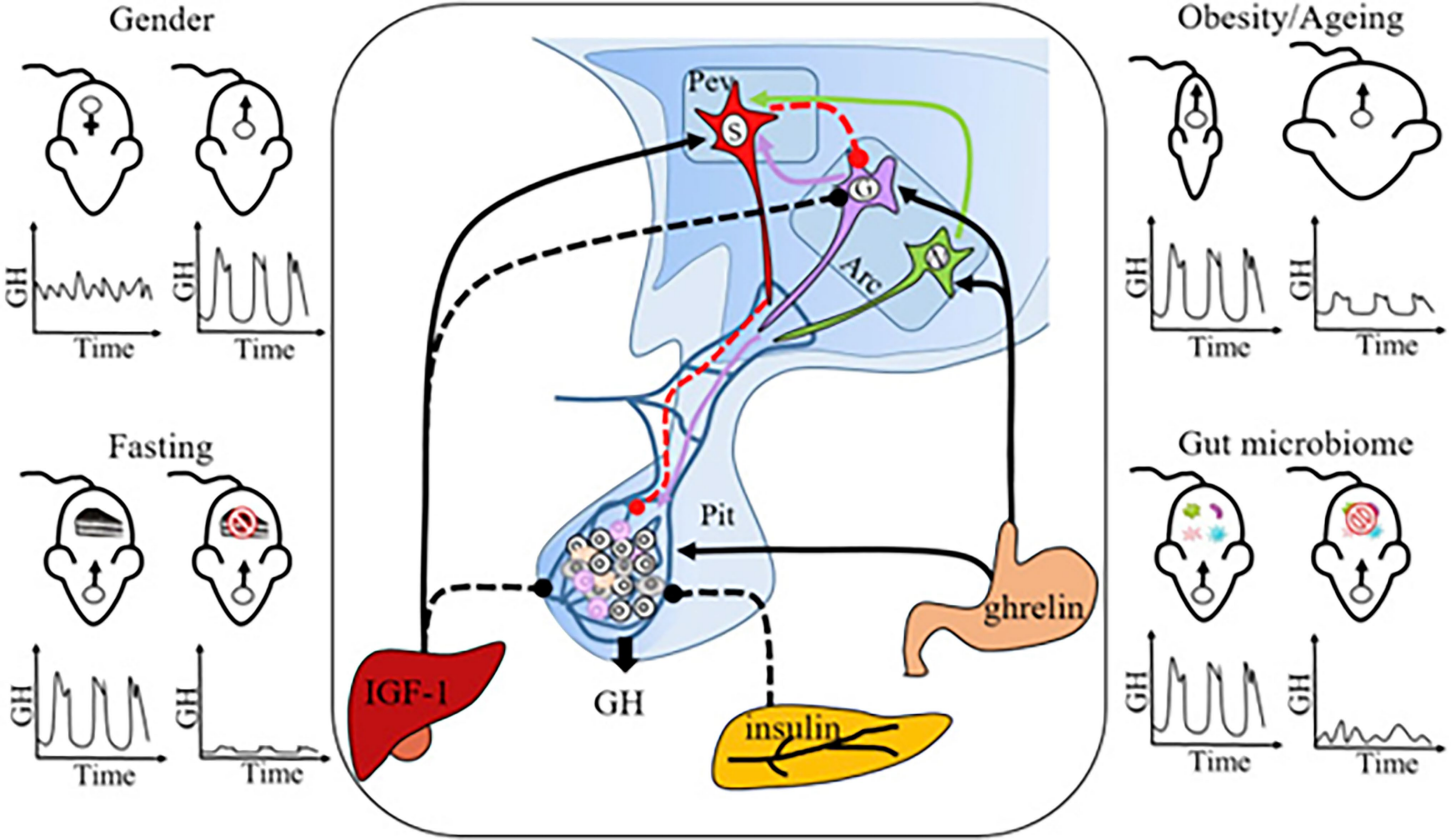
Neuro-Endocrine Mechanisms Regulating Sexually Dimorphic GH Secretion. Pulsatile GH secretion is directly regulated by stimulatory GH-releasing hormone (GHRH), expressed by neurons in the arcuate nucleus (Arc), and inhibitory somatostatin, expressed by neurons in the periventricular nucleus (Pev) of the hypothalamus. GH release is also regulated through feedbacks from peripheral factors, including IGF-1 (inhibitory), insulin (inhibitory), and ghrelin (stimulatory). Sexually dimorphic GH release is the normal physiological pattern (**top left panel**), but some challenges, including fasting (**bottom left panel**), obesity and aging (**top right panel**), and gut microbiome depletion (**bottom right panel**), feminize GH secretion. GH, growth hormone; Pev, periventricular nucleus; Arc, arcuate nucleus; Pit, pituitary gland; S, SRIF (somatotropin release inhibiting factor) neuron; G, GHRH neuron; N, NPY (neuropeptide Y) neuron; and IGF-1 = insulin-like growth factor-1. Solid lines: stimulatory effect; dashed lines: inhibitory effect. Figure used with permission from Huang et al. (2019).

GH pituitary release is regulated by two neuropeptide hormones, the stimulatory GH-releasing hormone (GHRH) produced by neurons in the hypothalamus, and the inhibitory somatostatin (SST) released by neurons in the pituitary (Figure 7). These two hormones influence pulsatile GH release by regulating each other’s secretion. GHRH stimulates GH release in the pituitary, which activates inhibitory signals from short-loop feedback inhibition (SST). Additional evidence that the hypothalamus–pituitary axis (HP axis) controls MUP production in the liver comes from a study on SST knockout (*Smst^−/−^*) mice: SST expression is greater in males than females, and *Smst^−/−^* male mice showed feminized hepatic MUP gene expression (which was not due to changes in T or T_4_ levels; Low et al., 2001). It turns out that this textbook model of GH regulation through two hormones is more complex than previously assumed, as several mechanisms regulate the HP axis and subsequent MUP excretion.

For example, ultradian GH secretion is paced by the circadian clock regulators (*Cryptochromes*, *Cry1* and *Cry2*), and double mutant male mice (*Cry1*^−/−^ *Cry2*^−/−^) lack a functional circadian clock and show female-like growth rates and body mass (Bur et al., 2009). Double mutants also have dramatically decreased *Mup1* gene expression and urinary MUP protein compared to controls (Bur et al., 2009). Sex differences in MUP gene expression were found to decline with aging (2-year-old C57BL/6 mice) due to altered GH profiles, which can be reversed by reinstating GH pulses in mutant *Cry*^−/−^ male mice. Deleting the circadian clock gene, *Bmal1*, disrupted the GH axis and reduced MUP expression (Schoeller et al., 2021), and *Mup2* expression is regulated by the circadian clock and glucocorticoids (Cho et al., 2011).

GH pulse rate is modulated by an array of neurotransmitters from the brain, including serotonin, acetylcholine, GABA, opioids (endorphins and enkephalins), and dopamine (Noaín et al., 2013; Ramirez et al., 2015; Brie et al., 2019), and also by peripheral hormones (long-loop feedback; Steyn et al., 2016; Le Tissier et al., 2018). For example, insulin-like growth factor 1 (IGF-1) provides negative feedback of GH release in the pituitary. GH stimulates IGF-1 synthesis in the liver (*via* the JAK2/STAT5 pathway), which negatively regulates GHRH and GH release (long-loop feedback inhibition). Knockout mice with liver-specific deletion of IGF-I (LI-IGF-I*^−/−^*) have low circulating IGF-1, which increases GH levels. Male LI-IGF-I*^−/−^* mice reduced urinary MUP output compared to controls, whereas the MUP output of female LI-IGF-I*^−/−^* mice was unaffected (Wallenius et al., 2001). Thus, disrupting the expression of IGF-I results in increasing GH levels (which is why it is used as a biomarker for pathological GH deficiency), and feminizing male MUP expression. However, experimental cGH administration does not significantly alter liver IGF-1 expression, unlike elevated GH from pathology (Lau-Corona et al., 2017). We are not aware of any evidence that physiological IGF-1 levels influence variation in MUP output, however. Some cytokines bind to GHRs in the liver, activate the JAK–STAT pathway, and then induce suppressor of cytokine signaling (SOCS) and CIS proteins that generate negative feedback and inhibit the signaling pathways that initiate their production (Matsumoto et al., 1999; Ram and Waxman, 1999; Krebs and Hilton, 2001). Deleting SOCS-2 genes (socs2^−/−^) disregulates GH signaling and reduces MUP levels in the urine (Metcalf et al., 2000). Other peripheral hormones that regulate GH secretion include insulin, leptin, ghrelin, nesfatins, and klotho (Devesa, 2021), though none have been shown to influence MUP production to our knowledge.

### Factors Affecting GH Pulsatile Secretion

Several studies have investigated various factors that influence GH signaling and sex differences in pituitary hormone release (Figure 7). The first measurements of GH pulsatile patterns in mice (Steyn et al., 2011) were obtained in a study of male (C57BL/6) mice housed at *ca*. 20°C and placed on an *ad libitum* diet. This study showed that one overnight fast resulted in a striking decrease in pulsatile GH secretion (reduced mass of GH secreted per burst, pulsatile and total GH secretion rate, and increased irregularity of GH pulses), whereas mean GH levels did not show a significant difference between fasted treatments versus controls. Subsequent studies have shown that fasting, obesity, microbiome depletion, and aging can feminize male GH secretion, but the underlying regulatory mechanisms are complex and still unclear (Huang et al., 2019). Some of these studies have also analyzed MUP expression. For example, an RNA-seq study found that germ-free mice have reduced levels of MUP genes and proteins in urine compared to controls (Weger et al., 2019). It was concluded sexually dimorphic MUP expression in the liver requires microbiota, which is likely due to their effects on sexual development and GH release.

Changes in male GH pulsatile release might also explain how acquiring dominant social status triggers increased urinary protein excretion in males but not females (Thoß et al., 2019; Luzynski et al., 2021). To our knowledge, no studies have investigated whether changes in social status or other behaviors influence GH pulsatile release, though GH *levels* have been found to influence behavior. A study on the male offspring of wild-caught mice examined changes in behavior following daily GH administration (Matte, 1981). Increased GH triggered isolation-induced aggression by reducing latency to fight and extending fighting duration. Another study compared the aggressive behavior of GH-sufficient males (heterozygous for the GHRH-KO allele) to homozygous knock-outs, when the mice were challenged with another male (Sagazio et al., 2011). The mice were divided in three groups: untreated controls, recombinant GH administration, or sham controls (with vehicle, Veh). The study found that the homozygous KO mice showed significantly reduced aggression compared to heterozygous males. GH (but not Veh) administration restored aggressive behavior of KO mice, despite not restoring serum IGF-I. There was no difference in serum T levels among these groups at any time. This study showed that GH-deficient males are less aggressive, that GH replacement normalizes aggressive behavior, and that these behavioral changes are not related to an increase in serum T. Thus, GH level can influence aggressive behavior of males, and studies are now needed to determine whether social status (winning fights or increased scent-marking) affect MUP production by influencing GH pulsatile release.

Although the mechanisms that regulate MUP expression in adult mice are becoming clear, it is still unclear how these mechanisms develop in early life.

## Ontogeny: Organizational Effects From Gonadal Steroids

Sexual dimorphic MUP expression was originally thought to be controlled solely by testosterone (T). Urinary MUP excretion begins at puberty (4–7 weeks of age), which is when serum T concentration begin to rise in males, and therefore, early studies on MUPs focused on measuring T. Castrated male mice were shown to excrete less urinary protein than intact males, and T-treated females excreted more protein compared to controls (Thung, 1956, 1962). Administrating T to females increased the amount of urinary protein they excreted and altered the electrophoretic pattern of these proteins (incidentally, the term “Major Urinary Protein” was first coined in these studies on mouse urine; Finlayson et al., 1963). T also influences the amount of MUP mRNA in the liver of castrated males and females (Osawa and Tomino, 1977; Szoka and Paigen, 1978; Hastie et al., 1979; Clissold et al., 1984). Implanting adult females with T increased hepatic MUP gene expression to levels similar to males, though the expression of some MUPs appeared to be more dependent upon T than others (Szoka and Paigen, 1978; Clissold et al., 1984), and it induced the excretion of MUP mRNAs with a male pattern (Knopf et al., 1983). T influences the expression of MUPs in the lacrimal gland, as well as in the liver (the only two tissues found to have male-biased expression in the study), but not in other tissues (Shaw et al., 1983). Another study found that serum T concentration was positively correlated with urinary protein excretion in male (CD-1) laboratory mice (Mucignat-Caretta et al., 2014), and studies are needed to confirm this result in naturalistic social conditions. It is unclear how T influences MUP production, though one possible mechanism is by programming the development of pathways in the hypothalamus and pituitary gland that control the release of growth hormone (GH).

Studies conducted on laboratory rats indicate that neonatal sex steroid hormones have organizational effects on the development of neural pathways and the GHRH and SST hormones in the hypothalamus that control ultradian GH secretion (Toews et al., 2021). Gonadal steroids continue to influence sex differences in GH secretory profiles during adulthood (Painson et al., 1992, 2000), which could explain the effects of T on MUPs (see above). The few studies on mice found similarities to rats. For example, administering T influenced hepatic MUP gene expression in adult mice by modulating the distribution of receptors of GHRH neurons (Bouyer et al., 2008). T also influenced the development of networks of GH cells in the pituitary that are dynamically regulated in adulthood (Sanchez-Cardenas et al., 2010). Administering T to neonatal females had organizational effect on the hypothalamus (GHRH) and IGF-1, as expected, however, exposure to T did not increase female hepatic MUP expression (Knopf et al., 1983; Ramirez et al., 2010). Thus, gonadal steroids may program and maintain sex-dimorphic patterns of the GH axis in mice, as with rats, but studies are still needed to clarify how gonadal hormones influence MUP production in mice.

## Adaptive Functions

The selective advantage of producing MUPs has long posed an interesting challenge to explain. The researchers who discovered MUPs were surprised to find so much protein in the urine of male mice, as protein in the urine is a pathological condition in humans (*uremia*). They were especially baffled to find that male mice synthesize tens of milligrams of protein per day in the liver, apparently only to excrete it. This seemed to be a “wasteful” and “irreversible loss” of protein. Subsequently, many studies have shown that MUPs provide a signaling mechanism for males to influence the brains and behavior of females. Sexual dimorphic traits are expected to evolve when traits have sex-specific fitness effects and generate intra-locus sexual conflict, but studies on how MUPs affect survival and reproductive success have only just begun.

### Chemosensory Signaling Functions: Sexual Selection

Most research on MUPs has focused on their chemosensory functions (Hurst and Beynon, 2004; Stopka et al., 2012; Mucignat-Caretta and Caretta, 2014). These studies have shown that MUPs influence male chemical signals and function as both pheromones and carriers of volatile pheromones. For example, MUP20, which is mainly expressed by males, attracts females and several volatile male pheromones are MUP ligands (Figure 1). In contrast, we are not aware of any studies that have shown that MUPs or MUP ligands influence female odor or its attractiveness to males. Studies on MUP-mediated chemical signaling, however, have often assumed that MUP expression is fixed, and ignored evidence that expression is phenotypically plastic (Table 1). There are also sex differences in the olfactory detection of MUPs, since MUP-detecting sensory neurons are selectively expressed in females during estrus (Dey et al., 2015). Different MUPs may have different roles on chemical communication, and the same MUP may have different signaling effects depending upon the sex of the sender and receiver. It is premature to make conclusions about sex differences in MUP-mediated chemical communication, however, because most studies have focused on males and comparable studies on females are lacking.

Male-biased MUP output is expected to be maintained by sexual selection on males for several reasons. First, the high levels of MUP output by males appear to mediate male–male competition for territories and access to females (intrasexual selection). Male house mice are more territorial and scent mark more than females, and male MUPs mediate aggressive interactions (Hurst and Beynon, 2004; Kaur et al., 2014). In seminatural populations, dominant territorial males have higher urinary protein output and MUP20 excretion than subordinates (Figure 5; Thoß et al., 2019; Luzynski et al., 2021). Some studies suggest that MUP output determines male social status, whereas others found that males upregulate urinary protein excretion after acquiring a territory (Thoß et al., 2019; Luzynski et al., 2021). Either way, elevated MUP output is expected to facilitate a male’s ability to defend a territory, which is a major determinant of male mating and reproductive success (Meagher et al., 2000; Luzynski et al., 2021). Females increase urinary protein excretion during aggressive female–female interactions (Garratt et al., 2011b; Stockley et al., 2013); but unlike male mice, dominant females in seminatural populations do not excrete higher levels of urinary protein compared to subordinates (Figure 5; Thoß et al., 2019; Luzynski et al., 2021).

Second, MUP output may also enhance male mating success through female mate choice (also called “intersexual selection,” which is an unfortunate term since the sexes are not competing). MUP20 in male urine influences female attractiveness to male odor, increases sex discrimination of scent marks, it induces spatial learning (Roberts et al., 2010, 2012), and after detection, it stimulates neural growth in the brain (Hoffman et al., 2015; Demir et al., 2020). Females are attracted to male urine spiked with MUPs when females are in estrus (Dey et al., 2015). Increased overall urinary protein concentration of males housed in laboratory conditions does not influence the attractiveness of females to male scent (Roberts et al., 2010), nor does it explain female preferences for the scent of dominant over subordinate males living in seminatural conditions (Thoß et al., 2019). Nevertheless, in natural conditions, high MUP output is expected to prolong the release of volatile male pheromones, increase the attraction of females to a male’s territory, and induce an acceleration of female puberty and estrous cycling (Mucignat-Caretta et al., 1995; Marchlewska-Koj et al., 2000; Morè, 2006; Flanagan et al., 2011).

These two types of sexual selection are not mutually exclusive, as female sexual preferences are influenced by the outcome of male–male competition: estrous females are more attracted to the urinary scent of dominant, territorial males, which excrete higher levels of urinary protein and MUP20 than subordinate males (Thoß et al., 2019). Thus, studies on chemical communication predict that MUPs mediate sexual selection in male mice and that MUPs are expected to enhance male mating and reproductive success.

In contrast, there is no evidence that MUP expression levels influence female odor, the outcome of female–female competition or their sexual attractiveness to males. Female urinary protein excretion increases just before and during estrus (Stopka et al., 2007), however, it is not known whether estrus-dependent MUP regulation influences female odor.

**Figure 8:**
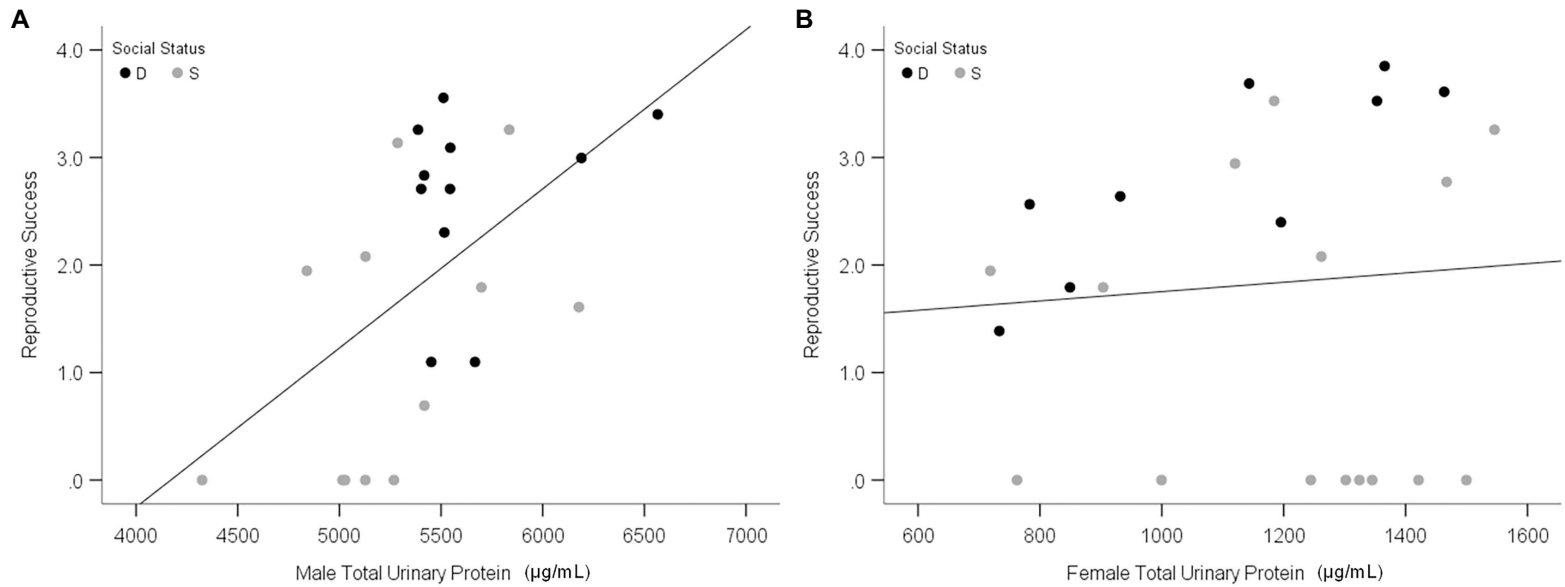
Relationship between urinary protein output and reproduction success. Scatterplots show the total urinary protein output of males **(A)** and females **(B)** in relation to individual reproductive success in the large, seminatural enclosures. Black and gray data points indicate dominant (D) and subordinate (S) social status, respectively. Reproductive success was calculated as the Ln (number of offspring per mouse + 1). Adapted from Luzynski et al. (2021).

Olfactory experiments on the attraction of females to male odor provide insights into the adaptive functions of MUP production, but they are insufficient to test hypotheses about sexual selection. Only one study to our knowledge has examined the effect of MUPs on reproductive success. This study found that urinary protein of wild-derived male mice (*M. musculus musculus*) is correlated with the reproductive success of males but not females (Figure 8; Luzynski et al., 2021) and that urinary protein excretion was the strongest correlate of male reproductive success. These findings support the hypothesis that male-biased MUP production is maintained by sexual selection in males. Studies are needed to determine whether these results are due to direct male–male competition, female mate choice, or both. Testing the mate choice hypothesis requires controlling for the effects of male–male competition (Thonhauser et al., 2013). Interactions between these two types of selection, however, make it difficult to examine their independent effects.

Studies are needed to explain why MUPs influence male but not female reproductive success, and why males regulate MUP output according to their social status, health, and condition (Table 1). To explain why males produce honest signals of their quality, it is often been suggested that MUPs function as a “handicap signal” (Malone et al., 2001; Stockley et al., 2013; Nelson et al., 2015). Zahavi’s Handicap Principle proposes that costly signals provide honest indicators of quality, not despite their costs, but because they are costly to produce. This hypothesis can be rejected for many reasons, however (Penn and Számadó, 2020). For example, it is illogical and it assumes that animal signals, unlike other traits, evolve under a non-Darwinian process of “signal selection” that favors waste rather than efficiency. The widespread acceptance of the Handicap Principle was due to Grafen’s (Grafen, 1990) “strategic choice” signaling model being misinterpreted as validating this idea, despite the fact that signals in this model are neither wasteful nor costly; on the contrary, they are efficient investments. It predicts that MUP output can provide honest signals of male social status, health, and other aspects of quality, if high-quality males have lower survival costs (or greater potential reproductive benefits) for producing MUPs than low-quality males. It is not known, however, whether male mice incur such differential fitness costs (or benefits) for MUP production.

It is often assumed that MUP production increases the absolute energetic costs of scent-marking, but the costs and benefits are likely to depend on a male quality or condition. Male MUP expression is condition-dependent (Table 1), suggesting that males in poor condition are less able to afford the energetic and other costs of producing MUPs. MUP production may *reduce* the net energetic costs of scent-marking of dominant males by reducing the effort, time as well as fitness costs (from predation and aggressive interactions) necessary to replenish territorial scent marks. However, almost nothing is known about how MUP production influences survival.

### Viability Effects: Metabolic and Other Physiological Functions

Only one study to our knowledge has investigated whether MUP production affects survival: MUP knockout mice (KO’s lacking MUP genes by deleting the entire 2.2 Mbp MUP gene cluster using CRISPR) were healthy for 2 years and did not show altered body mass or detrimental health effects compared to controls (Yang et al., 2016). This study was conducted in the laboratory, and therefore, studies are still needed to evaluate the fitness (longevity and reproductive success) of MUP-KO mice of both sexes living in more natural ecological conditions and exposed to physiological and other challenges.

Such experiments are crucial because MUPs provide physiological functions, including regulating metabolism (Petrak et al., 2007; Hui et al., 2009; Zhou et al., 2009) and eliminating harmful metabolic waste and toxic xenobiotics (Kwak et al., 2011, 2016; Stopka et al., 2016; Stopková et al., 2017; but see Nault et al., 2017). These findings are consistent with results from studies on the mechanisms that regulate MUP gene expression. GH controls the expression of many other genes in the liver that influence lipid and glucose metabolism and metabolism of xenobiotics. Their common regulatory pathways suggest that MUPs share common functions (“guilt by association”). Moreover, JAK2 and STAT5 deficiency results in hepatic lipid accumulation, and thus the regulatory proteins that control MUP expression may be crucial for survival in the wild. These findings can potentially help to explain the function of MUPs in female house mice, and why caged female mice elevate their MUP excretion after they are released from cages into naturalistic conditions (Thoß et al., 2019). And, if these physiological functions are more important for males than females, or more important for dominant than subordinate males, then this would help to explain differences in MUP gene expression between the sexes and among males. These results might also elucidate the original (ancestral) function of MUPs in rodents (see below), and help to explain the expansion of MUP loci in certain species (Charkoftaki et al., 2019). For example, multiple MUP loci might have enabled mice and rats, which are kleptoparasites (rather than commensals), to better cope with toxins when foraging on human refuse. No studies to our knowledge, however, have tested whether any of the proposed physiological functions of MUPs differ between the sexes, or whether they affect survival.

## Evolutionary Origins and Potential Effects on Divergence and Speciation

Determining the origins of sexually dimorphic MUP expression requires comparing MUP expression in both sexes among rodent species. The phylogeny MUP genes have been described (Stopka et al., 2012); however, few studies have compared urinary protein output (Nazarova et al., 2018) or MUP gene expression between the sexes in different *Mus* species (Sheehan et al., 2019; Matthews et al., 2021). Several studies have compared the MUPs of two European *Mus* subspecies and examined the hypothesis that divergence of these genes among populations promotes speciation.

**Figure 9:**
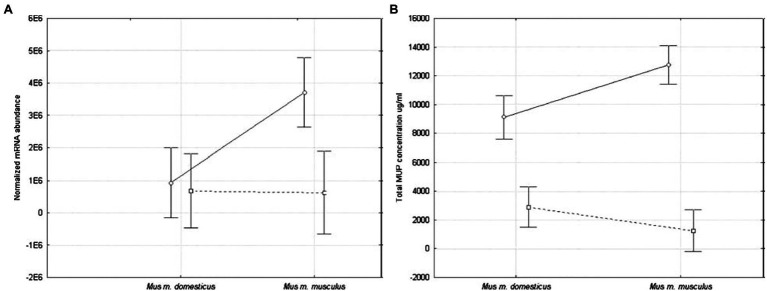
MUP expression in two *Mus musculus* subspecies. MUP output was compared using **(A)** normalized hepatic MUP mRNA abundance, and **(B)** concentration of urinary MUP protein circles connected by solid lines show differences between males, and squares connected by dashed lines show differences between females of these subspecies (error bars show 95% confidence intervals). Figure used with permission from Stopková et al. (2007).

The first study to provide a statistical comparison of MUP expression between *Mus* subspecies was conducted on wild-caught house mice from several populations near the European hybrid zone (Stopková et al., 2007). Quantitative differences between subspecies were found in hepatic MUP mRNA expression and total urinary MUP concentration (Figure 9). Male *M. musculus musculus* expressed more MUP mRNA than females, and more than *M. musculus domesticus* mice of either sex, and total urinary MUP concentration showed a larger sex bias in *M. m. musculus* than *M. musculus domesticus*. No differences were detected between females of these two subspecies.

**Figure 10:**
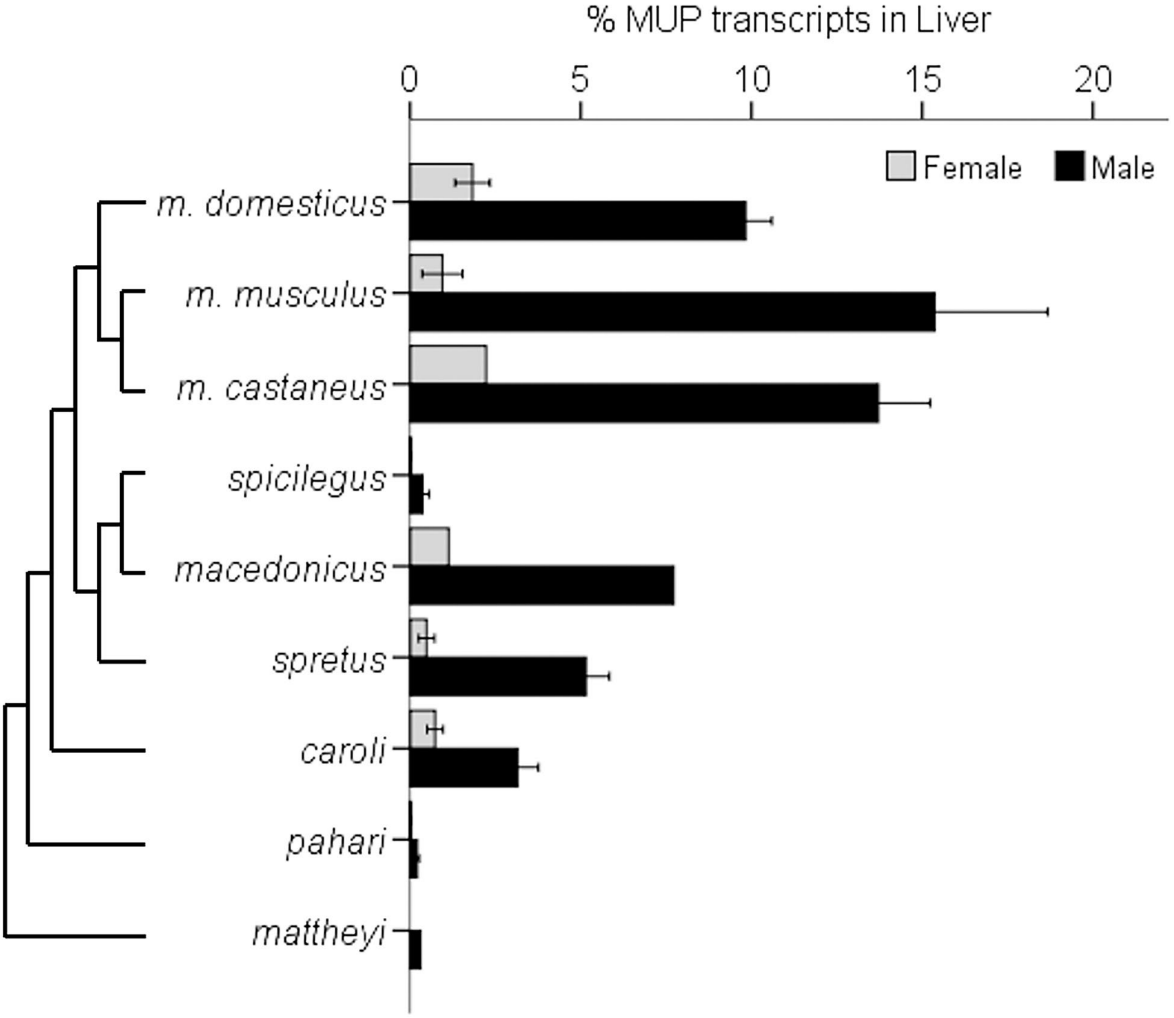
Sexually Dimorphic MUP Gene Expression in *Mus* Species. The graph illustrates sexually dimorphic expression of MUP genes in relation to a *Mus* species from Steppan and Schenk (2017) and *M. musculus* subspecific phylogeny from Macholán et al. (2012). Sex differences in MUP expression among *Mus* species and subspecies [mean percentages of total MUP gene expression in liver tissue composed of *Mup3, Mup20*, and central MUPs; data from Sheehan et al. (2019)]. Error bars are ±1 SEM (*N* = 1 where error bars are absent). *Mup20* is strongly male-biased in *M. musculus*, but it is not found in most other species, and although *Mup3* was thought to be male-specific, it is expressed in female *M. m. domesticus* and some other species. Data from Sheehan et al., (2019).

A recent comparative study found that the magnitude of sexual dimorphic gene expression varies among *Mus* species and subspecies (Sheehan et al., 2019). This study compared seven species of *Mus* and three *M. musculus* subspecies*. M. musculus* had the most pronounced sexually dimorphic MUP gene expression and protein output, as well as having more duplicated MUP loci, and *M. musculus musculus* showed the largest sexual dimorphism (Figure 10).

These results confirm that *M. m. musculus* show greater male-biased MUP expression than *M. m. domesticus* (Stopková et al., 2007; Figure 9) and other *Mus* species. They also indicate that sexually dimorphic MUP output of *M. musculus* is the derived rather than the ancestral trait, and that sexual dimorphism evolved by males increasing MUP expression rather than females reducing MUP expression. These findings raise questions about the underlying mechanisms explaining this pattern. For example, do *M. musculus musculus* males have higher rates of GH pulsatile secretion or sensitivity to GH pulsatile release than other species? They also raise questions about the ecological and social factors that correlate with species differences in MUP sexual dimorphisms. For example, are *Mus musculus* males more territorial or do they deposit more scent marks than males in other *Mus* species? To understand whether and how selection explains the evolution of sexually dimorphic MUP expression in some *Mus* species, studies are needed to experimentally test the effects of manipulating MUP expression of the fitness of both sexes in different species.

### Assortative Mating and Reinforcement

Stopková et al. (2007) suggested that differences in male MUP expression between subspecies might control subspecies recognition and explain assortative mating preferences in female *M. musculus musculus* observed in mice near the hybrid zone (Smadja and Ganem, 2002). Once perceptible differences in sexual characters evolve in diverging populations, and hybrids have reduced fitness, selection is expected to favor the evolution of assortative mating and further divergence and speciation (reinforcement hypothesis). In other words, the evolutionary divergence in male MUP expression among *Mus* species might drive further divergence between subspecies of mice through assortative mating.

Evidence for olfactory-mediated assortative mating preferences has been found in house mice trapped near the hybrid zone, and especially *M. musculus musculus* females (Smadja and Ganem, 2002). Yet, the mice were able to distinguish subspecies, regardless of whether the urine originated from mice in allopatric populations or the contact hybrid zone, suggesting that the divergence of the signal between subspecies originated in allopatry, contrary to the assumptions of the reinforcement hypothesis.

There have been several attempts to test the predictions of the MUP-mediated reinforcement hypothesis, including the following: (1) A genetic survey of wild house mice found considerable introgression between these two European subspecies for markers closely flanking the MUP cluster on both sides of a hybrid zone (Bímová et al., 2011), which contradicts assortative mating predictions. However, some alleles showed asymmetric introgression, as expected if only *M. musculus musculus* showed assortative preferences. (2) Another study scanned MUP and candidate olfactory (vomeronasal receptor or VR) genomic regions of mice from the hybrid zone and allopatric areas using microsatellite loci to detect recent selective sweeps (Smadja et al., 2015). Some MUP or VR loci displayed the expected reduction in variability in populations near the hybrid zone, but no strong conclusions could be made. (3) A detailed proteomic study compared the amount and types of urinary MUPs between these subspecies and their location near to the hybrid zones (Hurst et al., 2017). Urinary protein output was sexually dimorphic (overall males had *ca*. 3.5 times higher protein excretion than females), and *M. musculus musculus* had higher protein output than *M. musculus domesticus*; as previously shown (Stopková et al., 2007). Differences in male MUP excretion between subspecies were found in allopatric, but not contact zones, contrary to the reinforcement hypothesis. Subspecies divergence in the total urinary protein concentration in the contact zone was found in females, but not males due to the low concentration of protein in *M. musculus musculus* females. Several MUPs were expressed in one but not the other subspecies. The sample sizes were insufficient to make strong conclusions, however. (It was not possible to determine whether mass peaks that were shared between subspecies represent identical MUP isoforms due to the technical difficulties in resolving such highly homologous isoforms, even with peptide mass fingerprinting). The authors concluded that the differences in the expression profiles of urinary MUPs might have the potential to convey information about subspecific identity, and MUPs showing differential expression in the contact zone were suggested to provide candidates for assortative mating preferences and reinforcement.

No studies to our knowledge have tested whether MUPs mediate assortative mating, but if so, this hypothesis contradicts the proposal that house mice show disassortative mating preferences for MUPs (Sherborne et al., 2007). Thus, it is still unclear whether MUPs play a role in driving evolutionary divergence and speciation in house mice.

## Discussion

Here we summarize the key findings from studies on sexual dimorphic MUP expression and highlight questions that need to be addressed in the future. Our review shows that MUP production is generally male-biased, that most MUP genes have sexually dimorphic expression, and that are large differences in gene expression among MUP loci in both sexes. Moreover, we show that MUP output is male-biased in seminatural social conditions, as well as in the laboratory, and the regulation of MUP expression is more complex and dynamic in natural social contexts than in the laboratory (Enk et al., 2016; Thoß et al., 2019; Luzynski et al., 2021). MUP expression is not constitutive or fixed, and instead, it is regulated depending on a variety of different factors, including social status, caloric intake, infection, immune activation, and senescence, and males and females can differ in how they regulate MUP expression (Table 1). For example, male mice upregulate urinary protein output in response to acquiring a territory and dominant social status, unlike females (Thoß et al., 2019; Luzynski et al., 2021), and females regulate MUP excretion depending on their estrous stage (Stopka et al., 2007), which might explain why wild female mice show increased urinary protein excretion in more natural social conditions. More studies are needed on female mice in general, which is why funding agencies are beginning to require researchers to include both sexes in their grant proposals (Klein et al., 2015). Most studies on MUP expression have focused on *Mup1* and *20*, and research is needed to investigate the regulation of other MUP loci, which is challenging because MUPs are so highly homologous (Enk et al., 2016; Thoß et al., 2016), and longitudinal analyses are needed to determine whether MUPs are up- or downregulated (Thoß et al., 2019; Luzynski et al., 2021).

We also examined studies on the proximate mechanisms and evolution of sexual dimorphisms in MUP expression. First, studies on the proximate mechanisms controlling gene expression in the liver have shown that male-biased MUP gene expression is due to sex differences in GH pulsatile secretion from the pituitary, which induce MUP gene expression through the JAK2/STAT5 signaling pathway (Udy et al., 1997; Teglund et al., 1998; Holloway et al., 2008). Studies are still needed to confirm that normal physiological variations in GH secretion explain male-biased MUP output under more natural conditions, to determine how social status, caloric restriction, aging, and other factors male MUP production, and to compare MUP regulation in both sexes (Table 1). Many MUPs are expressed at low levels or silenced in females, and CUX2, a female-specific transcription factor, suppresses MUPs other sexually dimorphic genes in the liver (Conforto et al., 2012). GH regulates the expression of hundreds of other hepatic genes, but it is unclear whether their co-expression is inextricably linked or functional. It is also unclear how variation in GHR expression affects MUP synthesis, or how GHR expression is regulated. MUP expression is regulated by several hormones, and different MUPs are regulated by different endocrine mechanisms, but it is unclear how or why.

Second, GH release is controlled by neurons and hormones in the hypothalamus and pituitary, which appear to be organized by gonadal steroids, though studies are needed to explain their development (ontogeny).

Third, many studies show that male MUPs function as pheromones and pheromone carriers, though only two studies have investigated their fitness consequences: (1) One study found that increased levels of urinary protein excretion of wild mice living in seminatural conditions correlated with the reproductive success of males but not females (Luzynski et al., 2021). Studies are needed to determine whether this result is due to direct male–male interactions, female choice, or both. For example, estrous females are attracted to the scent of dominant territorial males, which excrete higher levels of MUP20 and several other MUPs compared to subordinates (Thoß et al., 2019; Luzynski et al., 2021). Taken together, these findings suggest that the regulation of MUP expression influences male reproductive success, such as through effects on the sexual attractiveness of urinary scent marks (Roberts et al., 2010, 2012) or other effects on the physiology and behavior of females (Hoffman et al., 2015; Demir et al., 2020). It is unclear why mice regulate MUP production (Table 1), and why males honestly signal their social status, health, and condition to rivals and potential mates; however, MUPs are not a “handicap signal.” It is often emphasized that MUPs are costly to produce, though MUPs may function to reduce the net costs, as well as to enhance the reproductive benefits of scent-marking by dominant males. (2) Another study examined whether MUP production influences survival, and though no effects were detected in MUP knockout mice of either sex, studies are needed to assess fitness effects in more natural conditions. MUPs provide physiological functions which may enhance survival, and perhaps more for males than females. If sexual dimorphisms are adaptive, then MUP expression should show differential fitness effects on the sexes, such that experimentally suppressing expression of males should impair their reproductive success, whereas elevating female MUP production to the levels of dominant males should reduce female survival or reproductive success. Such studies would help to test the hypothesis that sex differences in expression evolved due to intra-locus sexual conflict over allelic gene expression (Pennell and Morrow, 2013).

Fourth, studies have only just begun on the evolutionary origin of sexually dimorphic MUP expression in house mice. A recent comparative study of MUP gene expression in *Mus* species and *Mus musculus* subspecies (Sheehan et al., 2019). This analysis suggests that the ancestral state of MUP expression in *Mus* is sexually monomorphic, and that male-biased expression evolved by increasing male rather than reducing female MUP expression. However, the sample sizes of some species in this study were very small and housing conditions were not controlled. Studies are also needed to determine the underlying mechanisms controlling MUP gene expression and their functions in other *Mus* species. Comparative analyses are also needed to investigate whether sexual dimorphism co-evolved with the expansion of MUP loci in *Mus* and *Rattus*. MUPs could be used to test the hypothesis that sexual conflict favors the evolution of gene duplication (Cox and Calsbeek, 2009; Connallon and Clark, 2011; Gallach and Betrán, 2011). Other mammalian genera need to be investigated, as there are substantial differences in the sexual dimorphism in urinary protein excretion among different rodents, and house mice are not the most sexually dimorphic (Nazarova et al., 2018). It will be possible to begin reconstructing the evolutionary transitions that explain sexually dimorphic MUP expression once the genes that control MUP gene expression in *Mus musculus* and other *Mus* species are identified. It has been suggested that sex differences in MUP expression mediate assortative mating preferences, which subsequently drives further evolutionary divergence and speciation between house mice subspecies, but the evidence is mixed and direct tests are lacking.

## Author Contributions

DP wrote the first drafts, most text, and made most revisions. SZ and KL wrote the first draft of some sections and helped with the table, figures, and writing revisions and final editing. All authors contributed to the article and approved the submitted version.

## Funding

This work was funded by the Austrian Science Fund (FWF): P24711-B21 and P28141-B25. The funder had no role in study design, data collection and analysis, decision to publish, or preparation of the manuscript.

## Conflict of Interest

The authors declare that the research was conducted in the absence of any commercial or financial relationships that could be construed as a potential conflict of interest.

## Publisher’s Note

All claims expressed in this article are solely those of the authors and do not necessarily represent those of their affiliated organizations, or those of the publisher, the editors and the reviewers. Any product that may be evaluated in this article, or claim that may be made by its manufacturer, is not guaranteed or endorsed by the publisher.
